# NMR-based urinary biomarkers in pediatric primary mitochondrial disorders and chronic kidney disease: shared mitochondrial dysfunction, diverging biosignatures

**DOI:** 10.1007/s11306-025-02363-8

**Published:** 2026-01-19

**Authors:** Margarida Paiva Coelho, João E. Rodrigues, Teresa Costa, Aureliano Dias, Inês C. R. Graça, Hugo Rocha, Laura Vilarinho, Esmeralda Martins, Ana M. Gil

**Affiliations:** 1 Reference Center for Inherited Metabolic Disorders, Centro Hospitalar Universitário de Santo António, Unidade Local de Saúde de Santo António, Porto, 4099-001 Portugal; 2 Unit for Multidisciplinary Research in Biomedicine (UMIB), School of Medicine and Biomedical Sciences (ICBAS), University of Porto, Rua Jorge Viterbo Ferreira 228, Porto, 4050-313 Portugal; 3 Department of Chemistry and CICECO-Aveiro Institute of Materials, University of Aveiro, Campus Universitário de Santiago, Aveiro, 3810-193 Portugal; 4 Pediatric Nephrology Department, Centro Materno Infantil do Norte Albino Aroso, Centro Hospitalar Universitário de Santo António, Unidade Local de Saúde de Santo António, Porto, 4099-001 Portugal; 5 Newborn Screening, Metabolism and Genetics Unit, Human Genetics Department, National Institute of Health Doutor Ricardo Jorge, Lisbon, Portugal; 6 Department of Pathological, Cytological and Thanatological Anatomy, E2S, Polytechnic of Porto, Porto, Portugal

**Keywords:** Primary mitochondrial disorders, Pediatric CKD, Metabolomics, Urine, NMR, Biomarkers

## Abstract

**Background:**

Renal involvement is a recognized feature of primary mitochondrial disorders (PMD), either at presentation or during the disease course. Simultaneously, the metabolomic fingerprint of chronic kidney disease (CKD) is often associated with underlying mitochondrial dysfunction. This study aimed to characterize urinary metabolic signatures in genetically confirmed paediatric PMD without chronic kidney disease, comparing them to healthy controls, suspected (unconfirmed) mitochondrial disease (SMD), and non-mitochondrial CKD.

**Methods:**

We performed untargeted ^1^H NMR metabolomic profiling of 76 urine samples from 51 paediatric patients and 10 healthy controls. PMD patients in acute decompensation or known CKD and statistical outlier samples were excluded. Final comparisons included genetically confirmed PMD without CKD (*n* = 13), SMD (*n* = 10), non-mitochondrial CKD (*n* = 28; 17 at stages 1–2 and 9 at stages 3–5), and healthy controls (*n* = 10). Spectral data were analyzed using multivariate statistical approaches—including principal component analysis (PCA) and partial least squares–discriminant analysis (PLS-DA)—as well as univariate methods with Mann-Whitney U for pairwise group metabolite comparison.

**Results:**

Urinary metabolic profiles of PMD patients differed from healthy controls and CKD patients. Multivariate analysis revealed a strong discriminative ability between PMD and controls (Q² = 0.53) and advanced CKD (Q^2^ = 0.78). Compared to controls, PMD patients had increased levels of Krebs cycle intermediates (*cis*-aconitate, fumarate and succinate), creatine, tryptophan, homovanillate (HVA) and hypoxanthine, as well as decreased histidine. All, except fumarate and histidine, remained discriminative when comparing PMD to CKD. CKD patients showed a diverging metabolomic fingerprint with 1-methylnicotinamide (MNA) and 2-hydroxyisobutyrate emerging as potential CKD-specific biomarkers, effectively discriminating between CKD stage 3–5 from earlier stages and controls. A five-metabolite panel comprising *cis*-aconitate, fumarate, HVA, tryptophan and histidine achieved high diagnostic performance for identifying PMD, with an area under the curve (AUC) of 0.836 (PMD vs. controls) and AUC = 0.783 across all groups. This biosignature integrates metabolites involved in distinct functional domains including energy metabolism, neurotransmitter turnover and amino acid metabolism and renal handling.

**Conclusion:**

Urinary metabolomic profiling by NMR revealed a distinct biosignature in pediatric PMD patients without renal involvement, characterized by elevated levels of tryptophan, HVA, and Krebs cycle intermediates, and diminished histidine. The divergent changes in tryptophan, histidine and HVA, suggest a mitochondria-specific metabolic phenotype in PMD. These findings support the use of urinary NMR metabolomics as a non-invasive tool for biomarker discovery in PMD and highlight the potential of integrated, multiparametric metabolic fingerprints for diagnostic refinement and patient stratification.

**Supplementary Information:**

The online version contains supplementary material available at 10.1007/s11306-025-02363-8.

## Introduction

Renal involvement is a recognized and common feature of primary mitochondrial disorders (PMD), either as an initial manifestation or later in the disease course (Finsterer, 2022; Finsterer & Scorza, 2017; Hall et al., 2015; Parasyri et al., 2022; Roper et al., 2020). The most recognized renal phenotypes in PMD include proximal tubulopathy, nephrotic syndrome, focal segmental glomerulosclerosis and end-stage chronic kidney disease (CKD). Kidney involvement is also observed in conditions with secondary mitochondrial dysfunction conditions, such as Prader-Willi syndrome (Martín-Hernández et al., 2005).

Variants in both mitochondrial and nuclear-encoded mitochondrial genes are an established risk factors for kidney disease susceptibility and progression of CKD (Jotwani et al., 2024). Mitochondria play a central role in the pathophysiology of non-mitochondrial CKD and acute kidney injury, in an interplay between mitochondrial function and renal homeostasis (Bhatia et al., 2020; Duann & Lin, 2017; Galvan et al., 2017; Hu et al., 2018; Kazubek-Zemke et al., 2014; Kuhn et al., 2024; Rao et al., 2018). Metabolic profiling in CKD research has been extensively studied, having revealed impaired energy metabolism illustrated by elevated levels of Krebs cycle metabolites and diminished mitochondrial function and biogenesis, despite different CKD etiologies (Hao et al., 2013; Sharma et al., 2013; Posada-Ayala et al., 2014; Hong et al., 2019; Lucio-Gutiérrez et al., 2022). These profiles correlate with CKD stage and progression, underscoring the importance of mitochondrial dysfunction in renal pathophysiology (Kobayashi et al., 2014; Kimura et al., 2016; Khan et al., 2020; Lucio-Gutiérrez et al., 2022).

Urinary metabolite profiling, particularly through techniques such as ^1^H NMR or mass spectrometry (MS), has become an interesting non-invasive method for assessing mitochondrial dysfunction (Boenzi & Diodato, 2018). Only a few studies have comprehensively characterized the metabolomic profile of PMD patients, particularly in pediatric cohorts (Thompson Legault et al., 2015), most studies reporting targeted organic acid analyses or plasma-based metabolomics. Furthermore, until recently, some studies lacked genetic confirmation of diagnosis, and their findings must therefore be interpreted with caution (Smuts et al., 2010; Venter et al., 2015). More frequently, studies compared disease groups with healthy controls and less often with pre-symptomatic carriers, disease control groups (myopathy) or clinical control group (suspected not confirmed disease) (Barshop, 2004; Buzkova et al., 2018; Esterhuizen et al., 2017; Hall et al., 2015; Venter et al., 2015). For instance, the urine metabolome of adult mitochondrial patients with the m.3243 A > G variant showed significant differences from healthy controls, even in the absence of known renal involvement or relation to heteroplasmy levels, showing impaired redox balance, beta-oxidation, one-carbon metabolism, but also an altered trans-sulfuration pathway and methylation cycle(Esterhuizen et al., 2019; Hall et al., 2015).

To date, no studies have directly compared the urinary metabolomes of PMD and CKD patients, despite the growing evidence that renal dysfunction in mitochondrial disorders can mimic and coexist with CKD. In this context, untargeted ^1^H NMR metabolomics of urine may offer novel insight into disease-specific metabolic pathways and early renal involvement in PMD. This study aims to contribute to filling that knowledge gap by systematically comparing the urinary metabolomic profiles of pediatric patients with PMD, CKD, and SMD.

## Methods

### Subject recruitment and clinical and laboratory data collection

Patients were selected by the metabolic and nephrology pediatric teams based on availability and willingness to participate, without age or sex matching between groups. This study was approved by the local Ethics Committee, reference 82018.162(139-DEFI/138-CES) and written informed consent was obtained from all participants or their legal guardians, in accordance with the Declaration of Helsinki. As an observational study, no modifications to clinical management were made. This study analyzed urinary samples collected from pediatric patients followed at our center for suspected or confirmed mitochondrial or renal pathology, as well as healthy controls, between February 2020 and July 2024. A 2-year gap in recruitment occurred between March 2020 and September 2022 due to pandemic constraints. Inclusion and exclusion criteria, along with the recruitment process and sample numbers per group, are detailed in Table S1 and Figure S1.

Data extracted from clinical records included family history and detailed clinical presentation, encompassing neuromuscular symptoms, renal involvement, and other organ-specific manifestations. Regular medication use was also documented. Laboratory evaluation comprised blood counts, blood urea nitrogen (BUN), plasma and urinary creatinine, urea, and serum electrolytes (sodium, potassium, chloride, calcium). Urinary analyses included total proteinuria, microalbuminuria, α-1 and β-2 microglobulins, fractional excretion of sodium (FENa), tubular phosphate reabsorption, calciuria, glycosuria, urine pH, and specific gravity. Renal ultrasonography was performed in all patients with suspected or confirmed kidney disease. Patients were further classified according to the KDIGO 2024 guidelines, with glomerular filtration rate (GFR) estimated using the creatinine-based CKiD U25 Equation (or the average- creatinine-cystatin based U25 Equation when available), except for patients younger than 12 months, where estimated GFR (eGFR) was calculated using the Schwartz formula (Levin et al., 2024), and for patients older than 18 years, where eGFR was based on CKD-EPI 2021 equation. CKD severity was staged according to the 2024 KDIGO guidelines (Levin et al., 2024) and patients were stratified into two subgroups: stages 1–2 (mild CKD) and stages 3–5 (moderate to advanced CKD).

We recruited children from 2 months to 18 years of age into four groups (Table S2): (*i*) patients with genetically confirmed primary mitochondrial disorders including mutations in mitochondrial DNA (mtDNA) or nuclear genes (PMD, *n* = 15); (*ii*) individuals with clinical suspicion of mitochondrial disease but no established molecular diagnosis (SMD, *n* = 10); (*iii*) patients with chronic kidney disease (CKD, any stage, *n* = 29); and (*iv*) an opportunistic control group (*n* = 10) comprising otherwise healthy individuals followed in the Nephrology outpatient clinic, with no evidence of kidney or metabolic disease (e.g., mild vesicoureteral reflux or post-infectious follow-up). Regarding SMD, cases were selected based on suggestive clinical and biochemical features (e.g., elevated lactate and/or alanine, neuromuscular symptoms, developmental delay, radiological findings compatible with mitochondrial dysfunction) but without a confirmed genetic diagnosis. Only individuals with persistent suspicion of PMD and no alternative diagnosis after genetic testing were retained. In patients with chronic renal impairment of unknown etiology, mtDNA sequencing and whole exome sequencing (WES) was performed to exclude PMD. One patient with steroid-dependent nephrotic syndrome was reclassified as having a mitochondrial disorder related to *RRM2B*-related mitochondrial disorder (P25). Patients with PMD and known CKD phenotype were excluded (P25 and P15). Samples collected during acute decompensations or intercurrent infections were excluded based on clinical metadata and multivariate outlier detection, ensuring that profiles reflected patients’ baseline metabolic state. Out of the 64 patients initially screened, 58 were retained for final analysis (13 PMD, 10 SMD, 26 CKD patients and 10 controls) (Figure S1).

### Urine sampling

Casual urine samples were obtained during routine outpatient visits (therefore, irrespective of fasting period), promptly refrigerated, and aliquots were frozen (-80 °C) within 24 h. Clinical and general laboratory evaluations were performed on the same day. A single sample was collected per patient, except for 5 primary mitochondrial patients with known abnormal urinary metabolite excretion, for whom 2–5 samples were collected for time-variability assessment. In these cases, a single sample per patient was selected, minimizing factors such as acute exacerbations or disease progression (out of scope in this work).

### NMR spectroscopy

Urinary ¹H NMR analysis was performed on samples thawed at room temperature, after centrifugation, buffered with a phosphate solution (KH₂PO₄ in D₂O with 0.1% sodium salt of 3-(trimethylsilyl)propionic-2,2,3,3-d₄ acid, TSP), and pH adjusted to 7.40 (± 0.02) using KOH (potassium hydroxide, in D₂O) or HCl (hydrochloric acid, in D₂O). ¹H NMR spectra were acquired on a Bruker AVANCE III spectrometer operating at a frequency of 500 MHz for proton, using a standard NOESY-1D pulse sequence with pH adjustment and TSP calibration. Peak assignment was performed using Chenomx NMR Suite©, with the aid of 2D NMR spectra (total correlation spectroscopy- TOCSY and heteronuclear single quantum correlation- HSQC for selected samples) and of internal and public spectral libraries. A list of identified metabolites can be found in Table S3 (including some putative assignments, which would require additional spiking experiments, not carried out here). In some cases, statistical total correlation spectroscopy (STOCSY) (Cloarec et al., 2005) was used to aid metabolite assignment. Given the inclusion of patients with *TMEM70* and *TAZ*-related disease, typically associated with 3-methylglutaconic (3MGA) aciduria, we specifically searched for ^1^H NMR signals corresponding to previously reported 3MGA resonances (Engelke et al., 2006). No compatible resonance pattern was detected in our cohort, suggesting that 3MGA was either absent, below detection threshold, or not consistently elevated in basal conditions.

### Data preprocessing and statistical analysis

Spectral region exclusion was applied to eliminate regions corresponding to the water peak and the highly variable urea signal, which could introduce artefacts in multivariate analysis. Spectral alignment (Matlab R2014a, The MathWorks Inc., Natick, MA, USA) was performed prior to normalization through Probabilistic Quotient Normalization (PQN). PQN was chosen instead of total area normalization due to the large creatinine peak variations in several samples (reaching up to 12% of total spectral area in some cases). This rendered spectral total area not solely a function of sample dilution and, thus, not adequate for normalization in this study. Multivariate analysis (MVA) trough Principal component analysis (PCA) and partial-least-squares discriminant analysis (PLS-DA) models (SIMCA-P 11.5, Umetrics, Umeå, Sweden), were performed on full resolution ^1^H NMR spectra, after unit variance (UV) scaling of the spectra. MVA results were visualized through factorial coordinates (‘scores plots’) and factorial contributions (‘loadings plots’). The corresponding PLS-DA loading weights plots were back-transformed by multiplying each variable by its standard deviation and colored according to variable importance to the projection (VIP) (Matlab 8.3.0, The MathWorks Inc., Natick, Massachusetts, USA). PLS-DA models with predictive power (Q^2^) values ≥ 0.5 were considered of satisfactory or good robustness. Peak integration was performed after manual peak selection from the original spectra (Amix 3.9.5, Bruker BioSpin, Rheinstetten, Germany) and normalized using PQN. For comparison, normalization to creatinine was also carried out, due to still frequent use of this metabolite for concentration ratioing in the clinic, although acknowledging the inadequacy of this metabolite as a reference.

Individual metabolites´ variations were assessed using the Kruskal-Wallis test (for multiple group comparisons) and the Mann-Whitney U test (for pairwise comparisons), with a significance threshold of *p* < 0.05. Statistical analyses were performed using R software (version 4.3.2) and IBM SPSS Statistics (version 30.0). Metabolite variations were considered relevant if they exhibited a fold change (FC) ≤ 0.8 or ≥ 1.2 with a *p*-value < 0.05, or if they presented a significant effect size (Hedge’s g ≥ 0.2) with statistical significance. PLS-DA biplots for quantified peaks and corresponding VIP scores were generated using MetaboAnalyst 5.0, as well as, metabolites correlations, assessed with Spearman’s rank correlation coefficient. Significance Analysis of Metabolomics (SAM) and Empirical Bayesian Analysis of Metabolomics (EBAM) were also used to identify discriminant metabolites (supplementary material). Metabolic pathway analyses were based on the list of significantly altered metabolites, annotated using the KEGG database and literature review. Pathway enrichment and topology analyses were performed in MetaboAnalyst 5.0 using auto-scale data normalized to total spectral area. Analyses included KEGG pathway impact (topology and enrichment analysis) and metabolite set enrichment based on the SMPDB library.

To explore the diagnostic potential of urinary metabolites for PMD, we performed receiver operating characteristic (ROC) curve analysis and calculated the area under the curve (AUC) for each compound. A subset of candidate metabolites was then selected based on statistical significance (univariate tests), AUC thresholds, and multivariate variable importance. These selected metabolites were used to construct classification models—including logistic regression, PLS-DA, random forest, and linear support vector machines (SVM)—to assess their discriminative performance across diagnostic groups.

## Results

### Patients and group characteristics

Demographic and clinical characteristics across the five study groups are summarized in Table 1, including age and sex distribution, estimated glomerular filtration rate (eGFR), and microalbuminuria stages. The PMD group (*n* = 13) comprised patients with mtDNA mutations (*n* = 4), single mtDNA deletion (*n* = 1), and nuclear gene defects (*n* = 8), while all SMD cases (*n* = 10) remained undiagnosed. CKD groups were stratified into stages 1–2 (*n* = 17) and 3–5 (*n* = 9), with underlying diagnoses including glomerular disease, CAKUT, tubulopathies, and nephronophthisis. Significant albuminuria, according to KDIGO guidelines (A2: 30–300 mg/g; A3: >300 mg/g) were only observed in CKD groups, with A2 and A3 present in 59% and 41% of CKD1–2, and in 33% and 44% of CKD3–5, respectively. Detailed patient data are provided in Table S2.

**Table 1:** Group characteristics including sex and age distribution, estimated glomerular filtration rate (eGFR) and microalbuminuria categories, according to KDIGO guidelines (A2: ACR 30–300 mg/g; A3: ACR > 300 mg/g)

Group	*n*	Median Age [Range]	Sex (M/F)	eGFR mean ± SD	ACR stage A2 (%)	ACR stage 3 (%)	Diagnoses
Primary mitochondrial disease (PMD)	13	9.5 [1.1–18.8]	6/7	153.4 ± 63.2	0%	0%	mtDNA point mutations (*n* = 4) Single mtDNA deletion (*n* = 1) Nuclear gene defects (*n* = 8)
Suspected mitochondrial disease (SMD)	10	4.1 [0.2–18.7]	3/7	111.0 ± 18.7	0%	0%	Undiagnosed (*n* = 10)
Control	10	11.2 [2.7–18.2]	7/10	110.4 ± 9.2	0%	0%	CAKUT (isolated urogenital tract malformations) (*n* = 5) Mild vesicoureteral reflux (*n* = 2) Other (*n* = 3)
CKD stages 1–2	17	12.1 [2.8–17.9]	12/5	97.8 ± 15.3	59%	41%	Glomerular (*n* = 4) CAKUT (*n* = 3) Tubulopathies (*n* = 2) Other (*n* = 8)
CKD stages 3–5	**9**	12.9 [4.7–15.8]	6/3	20.3 ± 7.0	33%	44%	Glomerular (*n* = 3) CAKUT (*n* = 2) Nephronophthisis (*n* = 2) Other (*n* = 2)

Diagnoses were grouped into clinically relevant categories

*ACR* albumin-to-creatinine ratio, *eGFR* estimated glomerular filtration rate, *CKD* chronic kidney disease, *SD* standard deviation, *MA*, *mtDNA* mitochondrial DNA, *CAKUT* Congenital Anomalies of the Kidney and Urinary Tract

For group validation we performed supervised multivariate analysis (PLS-DA models) with full normalized spectra. PLS-DA was unable to discriminate between controls and the CKD stage 1–2 group (Q^2^ < 0) (Table S4), as anticipated by similar eGFR between groups and the recruitment strategy (opportunistic control group of children followed in the Nephrology clinic). To explore the impact of microalbuminuria as potential sources of variability within this cohort, we carried out additional subgroup comparisons comparing CKD stages 1–2 patients with and without significant microalbuminuria (ACR > 30 mg/g, regardless of its etiology). Discrimination was not significant (Q² = 0.29) (Table S4), supporting the clustering of all CKD stages 1–2 patients into a single group for downstream comparisons.

### General overview

**Fig. 1:**
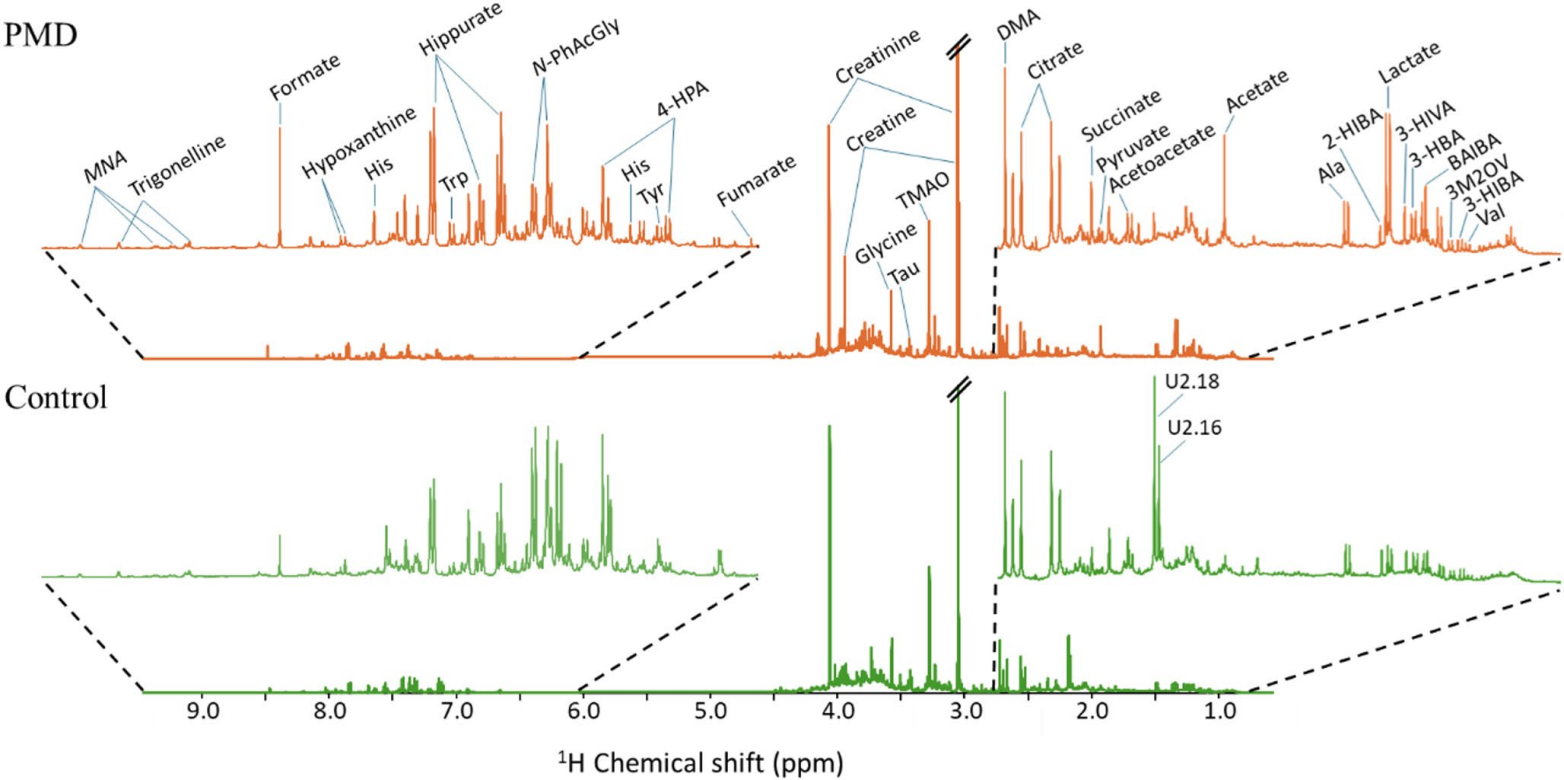
Average urinary ¹H NMR spectra of controls and patients with primary mitochondrial disease (PMD). Representative comparison between the average spectra of control subjects (green) and PMD patients (orange). Insets show expanded aliphatic (δ 0.8–2.8) and aromatic (δ 6.0–9.5) regions. Three-letter code used for amino acids. Abbreviations: 2-HIBA: 2-hydroxyisobutyrate; 3-HBA: 3-hydroxybutyrate; 3-HIBA: 3-hydroxyisobutyrate; 3-HIVA: 3-hydroxyisovalerate; 3M2OV: 3-methyl-2-oxovalerate; 4-HPA: 4-hydroxyphenylacetate; BAIBA: 3 (or beta)-aminoisobutyrate; DMA: dimethylamine; His: histidine; MNA: *N*-methylnicotinamide; *N*-PhAcGly: *N*-phenylacetylglycine; Tau: taurine; TMAO: trimethylamine *N*-oxide, Trp: tryptophan; Tyr: tyrosine. Compounds remaining unknown or not unambiguously assigned are represented by U followed by the corresponding ppm value

A comparative overview of the urinary metabolomic profile of average ¹H NMR spectra of controls and PMD patients is shown in Fig. 1, highlighted for regions 3.5–0.5 ppm and 9.5–6.0 ppm, revealed marked apparent differences in several chemical shift regions (Fig. 1). For instance, PMD samples seem to show, on average, higher levels of lactate, acetate, succinate, creatine, hippurate and formate, and possible lower levels of two unassigned resonances at 2.16 and 2.18 ppm. The latter may be putatively assigned to acetaminophen (either in its glucuronide and/or sulfate forms) methyls, as suggested by their strong statistical correlations (through STOCSY, results not shown) with several aromatic resonances (6.90, 7.10, 7.32, 7.42) as well as with a 5.14 ppm anomeric resonance confirming the glucuronide from. However, an unambiguous assignment would require further research, while at this stage no clear biological relevance to the study groups is identified. These visual changes refer only to average spectra and thus require further careful analysis through multivariate analysis and subsequent peak integration and univariate analysis.

Identified metabolites are summarized In Table S3. Overall, 57 metabolites were identified (4 of them still corresponding to putative assignments, requiring additional investigation to validate their identification). The identified metabolites are consistent with the literature for NMR urine metabolomics studies(Bouatra et al., 2013).

**Fig. 2:**
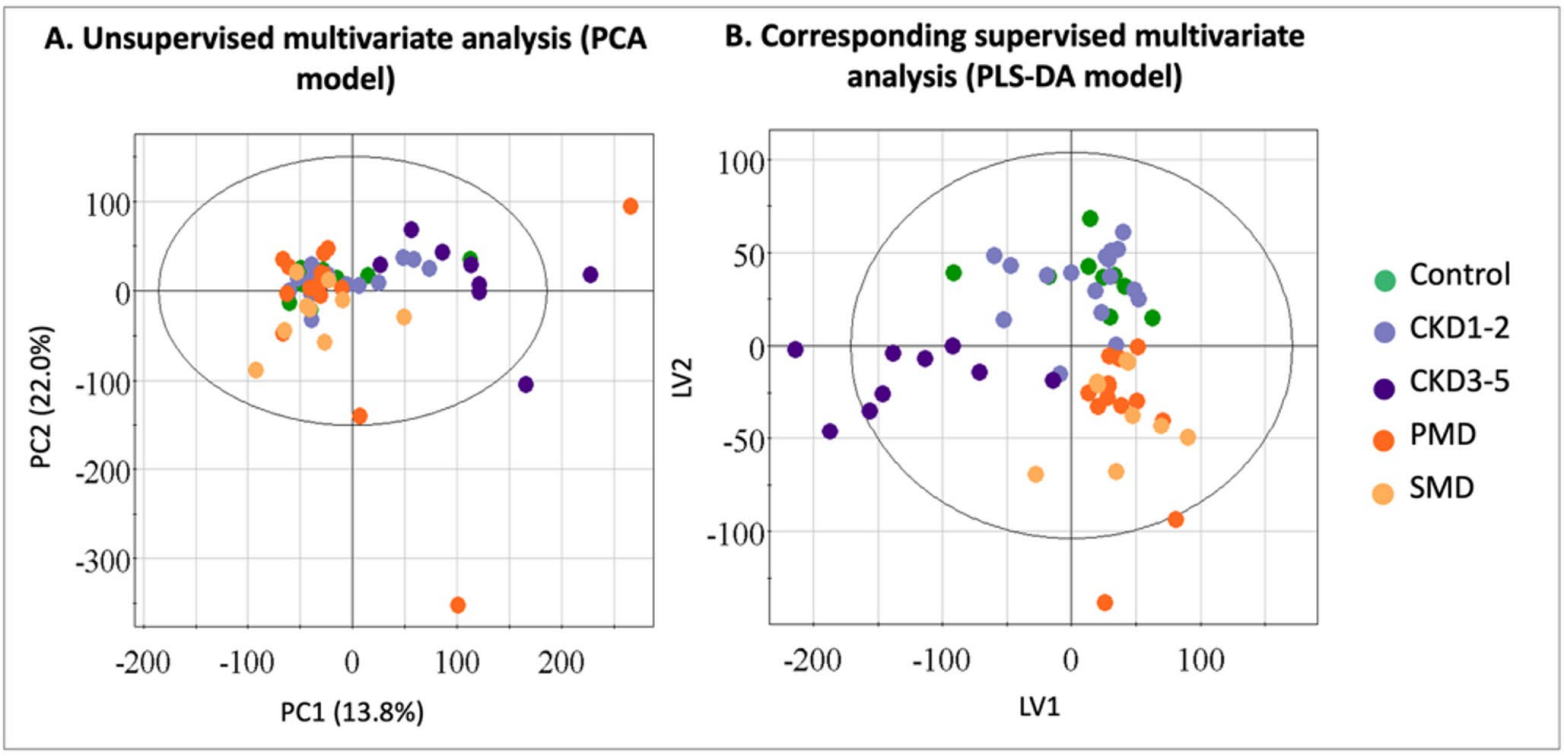
Multivariate analysis of urinary ¹H NMR metabolomic profiles across groups: Primary Mitochondrial Disease (PMD, orange), Suspected Mitochondrial Disease (SMD, yellow), Control individuals (green), Chronic Kidney Disease (CKD) stages 1–2 (lilac) and stages 3–5 (purple). The ellipses represent 95% confidence intervals. **A** Principal Component Analysis (PCA) highlights distinct metabolic fingerprint in CKD patients compared to other groups. **B** Corresponding supervised model using partial least squares discriminant analysis (PLS-DA) shows metabolic divergence between groups, particularly between PMD, CKD3-5, and controls. *PMD* primary mitochondrial disease, *SMD* suspected mitochondrial dysfunction, *CKD* chronic kidney disease

Unsupervised multivariate analysis performed by Principal Component Analysis (PCA) using all samples (Fig. 2A) highlighted distinct metabolic fingerprint in CKD patients compared to other groups. PLS-DA scores plot (Fig. 2B) illustrated a diverging tendency between groups, with the observation of three clusters: PMD and suspected mitochondrial disorders (SMD), CKD1-2 and Control, and CKD3-5.

**Fig. 3:**
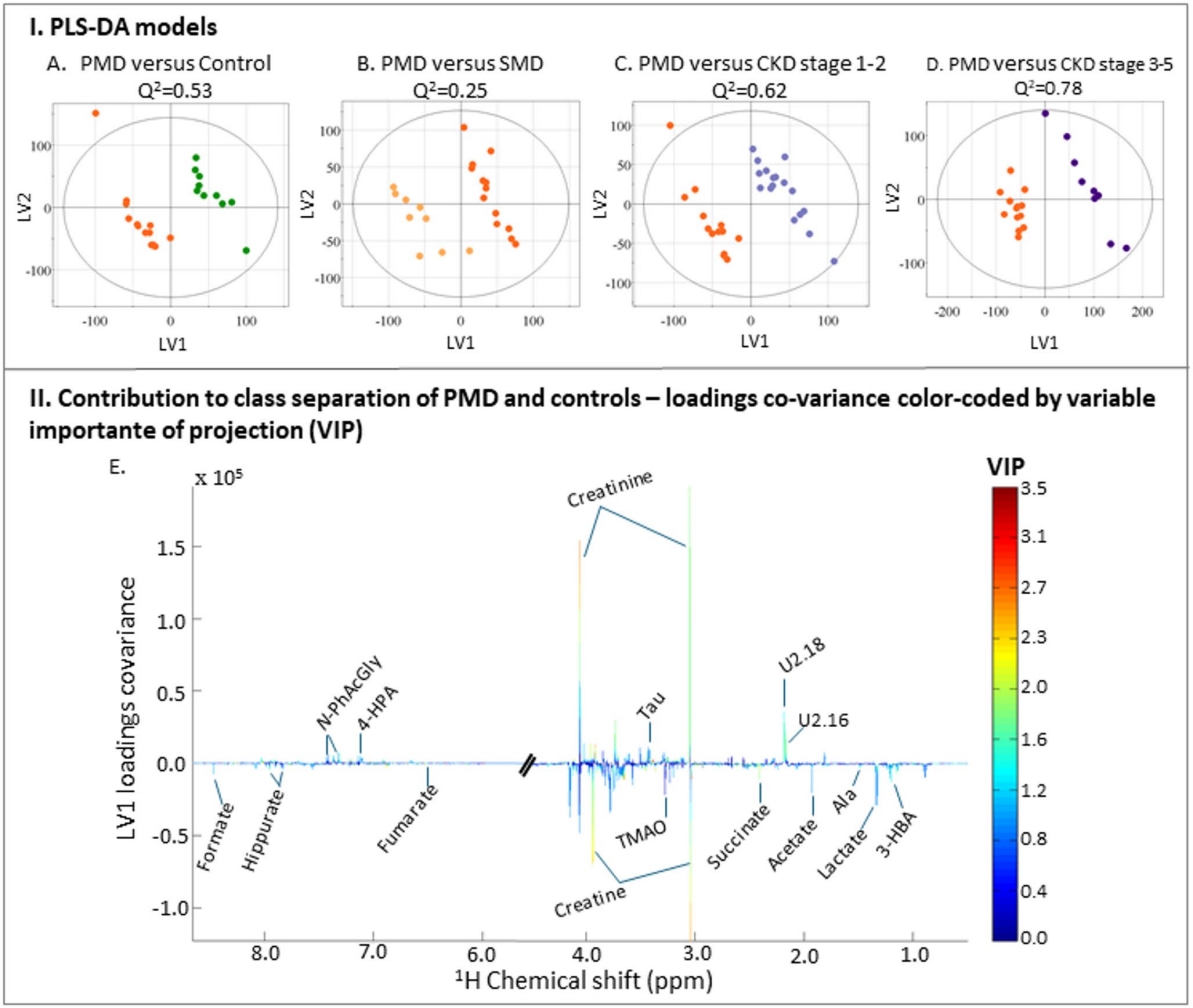
Supervised multivariate models and spectral features associated with mitochondrial disorders. I. Partial Least Squares Discriminant Analysis (PLS-DA) models were generated to compare patients with PMD to: **A** controls, **B** SMD, **C**. CKD stages 1–2, and **D**. CKD stages 3–5. The corresponding Q² values, indicate the predictive performance of each model. Clear discrimination was observed between PMD and controls (Q² = 0.53), and both CKD subgroups (Q² = 0.62 and 0.78). In contrast, the model comparing PMD to SMD showed lower predictive performance (Q² = 0.25), reflecting significant metabolic overlap. II. Corresponding loadings plot of model A, color-coded by variable importance in projection (VIP) scores for the first latent variable (LV1) highlighting spectral regions that contribute most to the separation between PMD and controls. (regions around 1.2–2.5 ppm and 3.0–4.5 ppm, consistent with metabolites involved in amino acid metabolism, organic acids, and energy-related pathways). Three-letter code used for amino acids. Abbreviations: 3-HBA: 3-hydroxybutyrate; 4-HPA: 4-hydroxyphenylacetate; *N*-PhAcGly: *N*-phenylacetylglycine. Ui: resonances at chemical shift i, which remain unassigned (or with tentative assignment)

Pairwise comparisons between groups (Fig. 3 and Table S4) confirmed the overall observed tendencies, with robust discrimination (Q² = 0.52) between PMD and controls (Fig. 3A). The corresponding loadings plot (Fig. 3E) revealed several resonances with high VIP values, suggesting higher amounts of organic acids (acetate, lactate, formate, fumarate and succinate), 3-HBA, alanine, creatine, hippurate and TMAO in PMD samples, and decreased quantities of 4-HPA, creatinine, *N*-PhAcGly and taurine, as well as two still unassigned peaks at 2.16 (U2.16, singlet) and 2.18 (U2.18, singlet), but possibly arising from acetaminophen forms, as mentioned earlier.

Despite the clear metabolic separation between PMD patients and healthy controls, multivariate analysis failed to discriminate PMD patients from individuals with SMD (Q² = 0.32). Regarding the other comparisons for PMD against the CDK group, vs. CKD1-2 (Fig. 3C) and vs. CKD3-5 (Fig. 3D), the PLS-DA models revealed a high predictive power (Q^2^ = 0.62 and Q^2^ = 0.78, respectively), reflecting strong metabolic differences between these groups.

To validate these findings, peak integration and corresponding statistical significance of metabolites variations we performed systematic pairwise comparisons using the Mann–Whitney U test for each metabolite. Quantified peaks are discriminated in Table S4. This approach was necessary given that omnibus tests (e.g., Kruskal–Wallis) may underestimate group-specific differences in large, heterogeneous datasets. Table 2 summarizes the urinary metabolites showing significant differences between PMD, CKD stage 3–5, SMD and control groups (Table S5 for remaining group comparisons). Fold changes (FC) and Hedge’s effect size (g) were calculated to assess group differences.

**Table 2:** Metabolite comparison across primary mitochondrial disease (PMD), control and chronic kidney disease (CKD) stages 3–5

Compound name	ppm	FC	Hedge’s (g)	*p*-value	Sign.^1^	
PMD vs. Control						
*cis*-Aconitate	3.11	1.48	0.63	0.020	*	
Creatine	3.93	1.84	0.95	0.020	*	
Fumarate	6.52	2.22	0.88	0.006	**	
Histidine	7.10	0.36	-0.62	0.038	*	
Homovanillate (HVA)	6.94	1.70	0.99	0.020	*	
Hypoxanthine	8.19	1.56	1.13	0.020	*	
Succinate	2.41	1.52	0.93	0.010	*	
Tryptophan	7.75	2.02	1.34	0.002	**	
PMD vs. CKD3-5						
1-Methylnicotinamide (MNA)	9.29	2.96	-0.95	0.001	***	
3-Aminoisobutyrate	2.20	1.42	-0.94	0.019	*	
2-Hydroxyisobutyrate	1.36	1.30	-0.76	0.033	*	
3-Hydroxybutyrate	1.23	1.59	-1.01	0.011	*	
3-Hydroxyisovalerate	1.27	2.08	-1.98	0.002	**	
4-Hydroxyphenylacetate	7.17	1.34	-0.39	0.045	*	
*cis*-Aconitate	3.11	1.68	-0.81	0.019	*	
Creatine	3.93	1.80	-1.01	0.033	*	
Formate	8.46	2.87	-0.62	0.006	**	
Glucuronate	5.27	0.09	1.23	0.003	**	
Glycolate	3.96	1.37	-0.83	0.038	*	
Homovanillate (HVA)	6.94	2.53	-1.79	0.001	***	
Hypoxanthine	8.19	1.46	-0.53	0.005	**	
Propylene glycol	1.14	2.31	-1.25	0.006	**	
Succinate	2.41	1.39	-0.86	0.045	*	
Tryptophan	7.75	1.73	-1.23	0.009	**	
Tyrosine	6.90	2.10	-1.26	0.001	***	
CKD3-5 vs. Control						
1-Methylnicotinamide (MNA)	9.29	0.35	-1.25	0.010	*	
3-Hydroxyisovalerate	1.27	0.42	-1.01	0.037	*	
Acetate	1.93	1.83	1.12	0.030	*	
Citrate	2.66	0.59	-0.71	0.004	**	
Fumarate	6.53	2.42	1.44	0.030	*	
Glucuronate	5.27	24.19	9.21	0.002	**	
Lactate	1.34	2.36	1.53	0.020	*	

Statistical comparisons were performed using Mann-Whitney U tests, normalized to PQN. Features with *p* < 0.05 but minimal variation (FC between 0.8 and 1.2) and negligible effect size (Hedge’s g between − 0.2 and 0.2) were excluded to avoid overinterpretation of biologically irrelevant findings

Shown chemical shifts correspond to the metabolite peaks used for integration. The table includes representative metabolites from amino acid metabolism, organic acids, and energy-related pathways, particularly those showing statistically significant differences between PMD and other groups, including controls and CKD stages 3–5

^1^ Statistical significance (Sign.): *p < 0.05, **p < 0.01, ***p < 0.001

*PMD* primary mitochondrial disease, *SMD* suspected mitochondrial dysfunction, *CKD* chronic kidney disease

### Urinary metabolomic of primary mitochondrial disease differs from controls with minor differences to suspected mitochondrial disease

Several metabolites displayed moderate to large effect sizes in the comparison between patients with PMD and controls. Krebs cycle metabolites (*cis-*aconitate, succinate and fumarate) were significantly elevated in the PMD group (FC 1.48–2.22, *p* < 0.02), with moderate to large effect sizes (Hedges’ g = 0.63–0.93). Tryptophan, hypoxanthine, and homovanillate (HVA) were significantly increased in PMD compared to controls (fold change 1.56–2.02), all showing large effect sizes (Hedges’ g = 0.99–1.34), supporting their potential as discriminant metabolic markers. For the latter, considering the existence of a significant outlier, we repeated the Mann–Whitney U test after excluding it, and the difference remained statistically significant (*p* = 0.030), confirming that the result was not driven solely by a single outlier. Histidine was the only significantly reduced metabolite in PMD (FC = 0.36, g = − 0.62). The EBAM analysis identified tryptophan, fumarate, succinate, hypoxanthine, and *cis*-aconitate as the most statistically robust metabolites distinguishing PMD from controls, based on the lowest local false discovery rates and highest posterior probabilities. These findings corroborate the Mann–Whitney results and highlight the biological relevance of these metabolites.

PLS-DA biplot and Volcano Plot based on quantified metabolites (one peak per metabolite) between PMD patients and controls, allows a visualization of the direction and relative contribution of individual metabolites to group discrimination and corresponding VIP plot listing the top discriminating metabolites per group (Figure S2). Correlation analysis revealed multiple clusters of positively correlated metabolites with shared biochemical roles (Figures S3). Fumarate was most strongly correlated with other Krebs cycles intermediates but also with HVA, tryptophan, isoleucine and tyrosine. *cis*-Aconitate displayed strong associations with HVA, 3-hydroxyisobutirate and 2-hydroxyisovalerate. Histidine showed weak to moderate negative associations with hypoxanthine and tryptophan. These findings suggest coordinated metabolic responses within mitochondrial-related pathways, with specific signatures centered around Krebs cycle intermediates and amino acid derivatives.

Pathway enrichment analysis (SMPDB and KEGG-based analysis) revealed multiple significant perturbations in mitochondrial-related and amino acid metabolism pathways in PMD patients compared to controls (Figures S3 and S4). The most relevant pathways included the as expected the energy metabolism and redox balance - pyruvate metabolism, Krebs cycle and mitochondrial electron transport chain and beta-oxidation. Additional relevant pathways included oxidation of branched-chain fatty acids, glutamate, arginine, proline, tryptophan metabolism, and glutathione metabolism.

While Krebs cycle intermediates, such as *cis*-aconitate, fumarate, and succinate, and creatine were elevated in both PMD and SMD relative to controls, the associated fold changes and effect sizes were consistently lower in the SMD group (e.g., FC for fumarate: 1.99 in SMD vs. 2.22 in PMD). These metabolites did not differ significantly between PMD and SMD, indicating overlapping metabolic profiles. In addition, 3-hydroxybutyrate (FC = 1.23, *p* = 0.045) and propylene glycol (FC = 1.69, *p* = 0.028) were significantly increased in PMD compared to SMD but did not differ significantly from controls in either group.

Tryptophan was significantly increased in PMD compared to controls (FC = 2.02, *p* = 0.002), while no significant change was observed in SMD (FC = 0.91, *p* = 0.055); the PMD and SMD levels were similar (FC 1.18) yielding a non-significant difference. Histidine was significantly reduced in PMD relative to both controls (FC = 0.36, *p* = 0.038) and SMD (FC = 0.50, *p* = 0.005), with no significant difference between SMD and controls, suggesting a discriminatory role for this metabolite.

### Diverging urinary metabolomic signatures between chronic kidney disease and mitochondrial disorders

The urinary metabolome of advanced CKD (stages 3–5) differed significantly from controls and early CKD (stages 1–2). Significant increases were observed for lactate, fumarate and acetate, while citrate and 1*-*methylnicotinamide (MNA) were reduced. Glucuronate levels were markedly elevated when compared to controls and CKD1-2 (FC = 24.19 and 6.69, respectively).

However, the urinary metabolome of PMD differed markedly from patients with CKD across stages (Q² >0.62), particularly with advanced CKD (stages 3–5). Seven teen urinary metabolites (Table 2) showed significant differences between PMD and CKD stages 3–5, including Krebs cycle intermediates (*cis*-aconitate and succinate), as well as 3-hydroxyisovalerate, 2-hydroxyisobutyrate, tryptophan, tyrosine, and HVA, all showing large effect sizes (Hedges’ g > 1), consistent with strong discriminatory potential. Additionally, the levels of formate, MNA, and propylene glycol were elevated in PMD, whereas that of glucuronate was markedly reduced (FC = 0.09, g = 1.23).

**Fig. 4:**
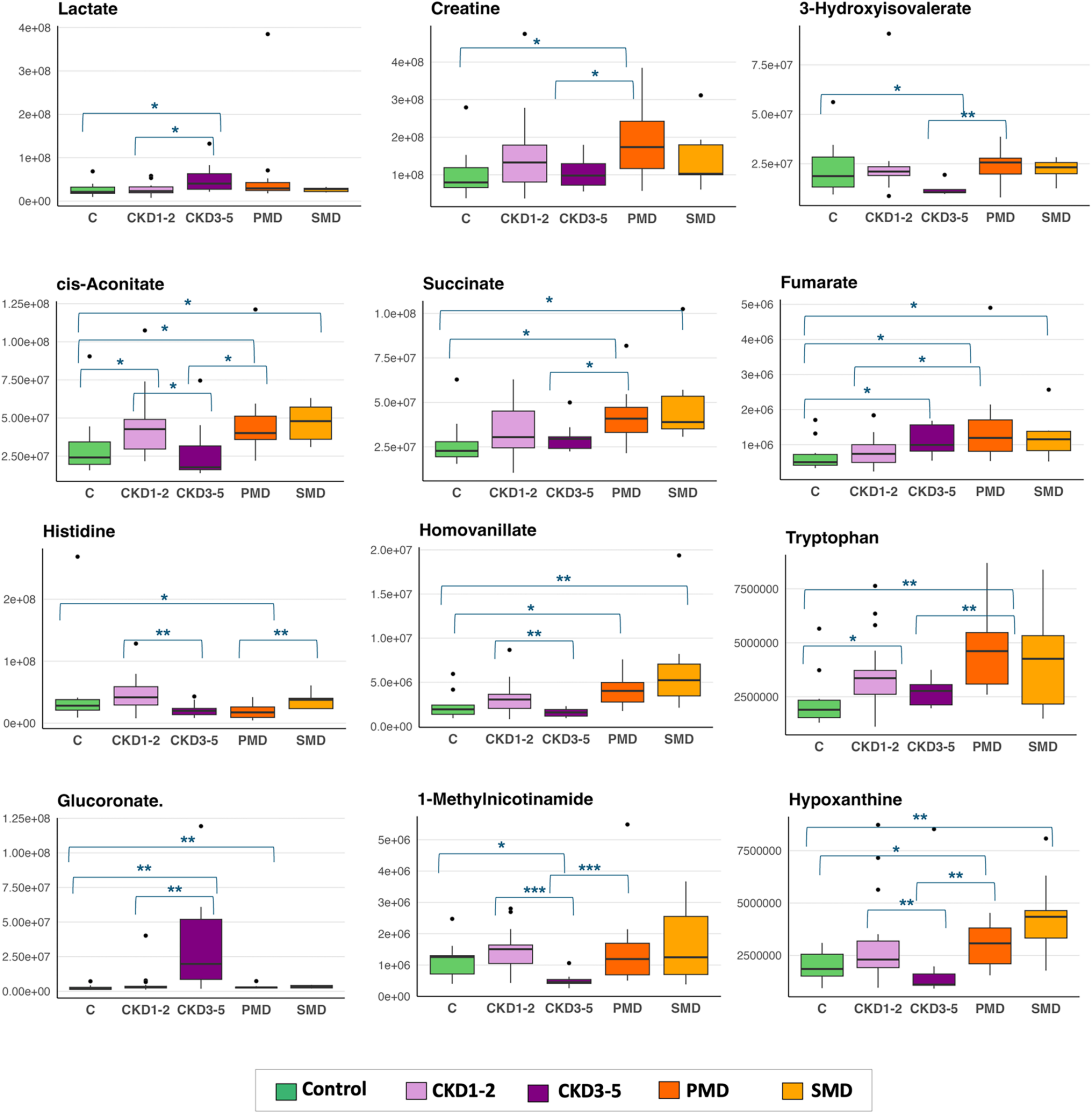
Boxplots of selected urinary metabolites showing statistically significant differences across primary mitochondrial disease (PMD), control, suspected mitochondrial dysfunction (SMD), and chronic kidney disease (CKD) groups at stages 1–2 and 3–5. Metabolites were selected based on biological relevance and statistical significance (non-parametric Mann-Whitney U test for each comparison). Significance thresholds are indicated as *p* < 0.05, **p < 0.01, ***p < 0.001. Corresponding statistical results are presented in Table 2 (main group comparisons) and Table S5 (remaining group comparisons). *PMD*, primary mitochondrial disease; *SMD*, suspected mitochondrial dysfunction; *CKD*, chronic kidney disease

In fact, several metabolites showed with divergent profiles between PMD and CKD3-5 with opposite directions of change relative to controls and significant differences between the two groups (Fig. 4). Among divergent metabolites, HVA, hypoxanthine, succinate, tryptophan, were significantly elevated in PMD compared to both CKD3-5 and controls and histidine was selectively decreased in PMD, with no significant change in CKD3-5. Conversely, MNA and 3-hydroxyisovaleric acid were significantly reduced, and glucuronate strongly increased, in CKD3-5 but not in PMD, pointing to renal-specific metabolic alterations likely reflecting impaired excretion or tubular dysfunction.

Decreased MNA and 2-hydroxyisovalerate consistently distinguished CKD from controls and further discriminated CKD3–5 from CKD1–2 (Fig. 4), showing progressive depletion with advancing renal dysfunction, which is according to previous studies(Kwan et al., 2020; Lucio-Gutiérrez et al., 2022). These patterns support their potential as specific biomarkers markers of CKD disease and potential correlation with stage severity.

### Impact of normalization strategy on urinary metabolomic profiles

To assess the impact of normalization strategies on metabolomic data, both PQN and to urinary creatinine (a common strategy in clinical setting) were applied and their consistency was computed by Spearman’s rank correlation coefficients for each quantified metabolite (Table S6). Overall, the correlation of 38 metabolites between the two approaches was moderate (median *r*_*s*_ = 0.48), with only 5 metabolites exhibiting strong or very strong monotonic relationships (*r*_*s*_ ≥ 0.7) while 10 had no correlation (*r*_*s*_ < 0.3). However, a 10% of analytes showed weak (*p* = 0.1–0.3) or no correlation (*p* < 0.1), including among others, urea, inosine, glycolate and DMA. For CKD-related univariate comparisons (114 pairwise metabolite comparisons) 75% showed concordant statistical significance under both normalization strategies. However, 20 comparisons were statistically discordant − 15 of which emerged when comparing CKD stages. Only a small subset of metabolites (including acetate, pyruvate, trigonelline, TMAO, and MNA) reached statistical significance exclusively under creatinine normalization. In our study this impact was most pronounced in comparisons involving CKD groups, particularly stages 3–5, where markedly reduced urinary creatinine concentrations contributed to a loss of statistical significance for several metabolites under creatinine normalization (such as 3-hydroxybutyrate, histidine, HVA, tyrosine, formate, hypoxanthine or tryptophan).

While normalization to urinary creatinine is widely used in clinical practice to adjust for variations in sample concentration, these results show that the method may introduce systematic *bias* in pediatric or pathophysiological settings where creatinine excretion is altered, such as CKD and myopathy (common in PMD). In contrast, PQN-normalized NMR data offer a data-driven alternative based on the overall spectral profile and is less influenced by single-analyte variability. In any case, in our cohort, measured urinary creatinine (by photometric assays) was significantly decreased only in the CKD3–5 group, which would attenuate group differences specifically in this subset.

### Candidate biomarkers for PMD

ROC analysis supported the discriminative potential of several urinary metabolites for PMD. Histidine (AUC = 0.78, log2FC = − 1.15) and tryptophan (AUC = 0.74, log2FC = + 0.56) showed the highest specificity for PMD versus other groups, aligned with univariate findings. Propylene glycol, creatine, and fumarate also showed good performance (AUC ≥ 0.71), reflecting mitochondrial dysfunction, although with less specificity.

A targeted five-metabolite panel consisting of *cis*-aconitate, fumarate, HVA, tryptophan and histidine achieved significant separation across all five diagnostic groups (PMD, SMD, CKD1–2, CKD3–5, controls) in one-way ANOVA and displayed strong classification performance in Random Forest analysis. These metabolites contributed consistently to group discrimination, supporting their potential as a biosignature for diagnostic stratification.

To further assess the potential clinical applicability, of the above metabolite signature, we constructed classification models using the most discriminative compounds. The same, five-metabolite panel achieved excellent accuracy in distinguishing PMD from controls, with AUCs of 0.949 (linear SVM), 0.938 (PLS-DA), 0.88 (random forest), and 0.836 (logistic regression). When applied to a broader comparison of PMD versus all others, it maintained strong discriminative power (logistic regression AUC = 0.783), highlighting its robustness in non-binary diagnostic classification.

## Discussion

To our knowledge, no previous metabolomic studies have directly compared urinary profiles between PMD and CKD. The use of disease-specific control groups enables a more precise attribution of metabolic changes to disease-specific mechanisms, reducing confounding from overlapping or nonspecific differences. This comparison is particularly important because renal dysfunction can mimic or coexist with mitochondrial phenotypes, independently affecting the excretion of metabolites typically interpreted as mitochondrial markers. Focusing on younger patients minimizes the influence of age-related comorbidities.

The normalization strategy is a critical step in urinary metabolomics to ensure comparability across samples. Normalization to urinary creatinine, a conventional approach in clinical setting, assumes stable endogenous creatinine excretion. However, creatinine is lower during infancy (under one year of age, as in patients from our SMD group) and in patients with myopathy, or impaired renal function (due to reduced glomerular filtration). In our study this impact was most pronounced in comparisons involving CKD groups, particularly stages 3–5, where markedly reduced urinary creatinine concentrations tends to underestimated changes.

### Distinct metabolomic signatures in PMD patients

Our findings reveal that the urinary metabolome of pediatric PMD patients is significantly different from healthy controls, even under basal clinical conditions. Differences were consistently observed in multivariate and univariate analyses, supporting the existence of a mitochondrial disease-specific metabolic footprint.

Key metabolic alterations in PMD included increased urinary levels of HVA, *cis*-aconitate and fumarate and decreased histidine with moderate-to-large effect sizes compared to controls and CKD stage 3–5 patients, revealing how differently PMD and CKD impact the urinary metabolome. Pathway enrichment analysis identified significant involvement of energy metabolism and redox balance, including glutathione metabolism, as well as pathways related to amino acid turnover—specifically glutamate, arginine, proline, and tryptophan metabolism—and the oxidation of branched-chain fatty acids. These findings support the biological consistency of the urinary metabolomic profile in PMD and underscore the contribution of mitochondrial energy production, amino acid catabolism, and oxidative stress response to its pathophysiology.

Substantial overlap was observed between PMD and SMD and effect sizes were generally small. These findings reflect the heterogeneity of the group and probably reflect selection bias for patients with biochemical evidence of mitochondrial dysfunction to be considered in this group. It most likely includes both true mitochondrial disorders and “mitochondrial mimics”.

### Mitochondrial impairment in renal disease differs from primary mitochondrial dysfunction

Despite the shared involvement of mitochondrial pathways in both PMD and CKD, our data support the existence of distinct urinary metabolomic signatures. In PMD patients compared to controls, elevated levels of fumarate, *cis*-aconitate tryptophan and HVA were consistently observed, even in the absence of renal impairment. In contrast, these metabolites were either unchanged or variably modulated in CKD, with *cis*-aconitate showing an inverse trend with CKD progression. CKD3–5 patients exhibited increased urinary excretion of MNA, 3-hydroxybutyrate, and other branched-chain organic acids, alongside reductions in HVA and histidine.

These findings emphasize that while mitochondrial dysfunction is a recognized feature of CKD pathophysiology, the metabolic footprint of CKD differs significantly from that of primary mitochondrial disease. Notably, many of the altered metabolites are linked to mitochondrial energy metabolism (e.g., succinate, formate, 3-hydroxybutyrate), amino acid turnover (e.g., tyrosine, tryptophan, hypoxanthine), or renal handling of organic acids (e.g., glycolate, glucuronate). Among Krebs cycle intermediates, fumarate was the only metabolite significantly increased in CKD3-5, suggesting an altered mitochondrial or renal tubular handling. In contrast, citrate and *cis-*aconitate levels were markedly reduced, and succinate showed only non-significant trends toward elevation, reinforcing fumarate’s potential as a CKD-associated metabolite with preserved detectability in urine.

**Fig. 5:**
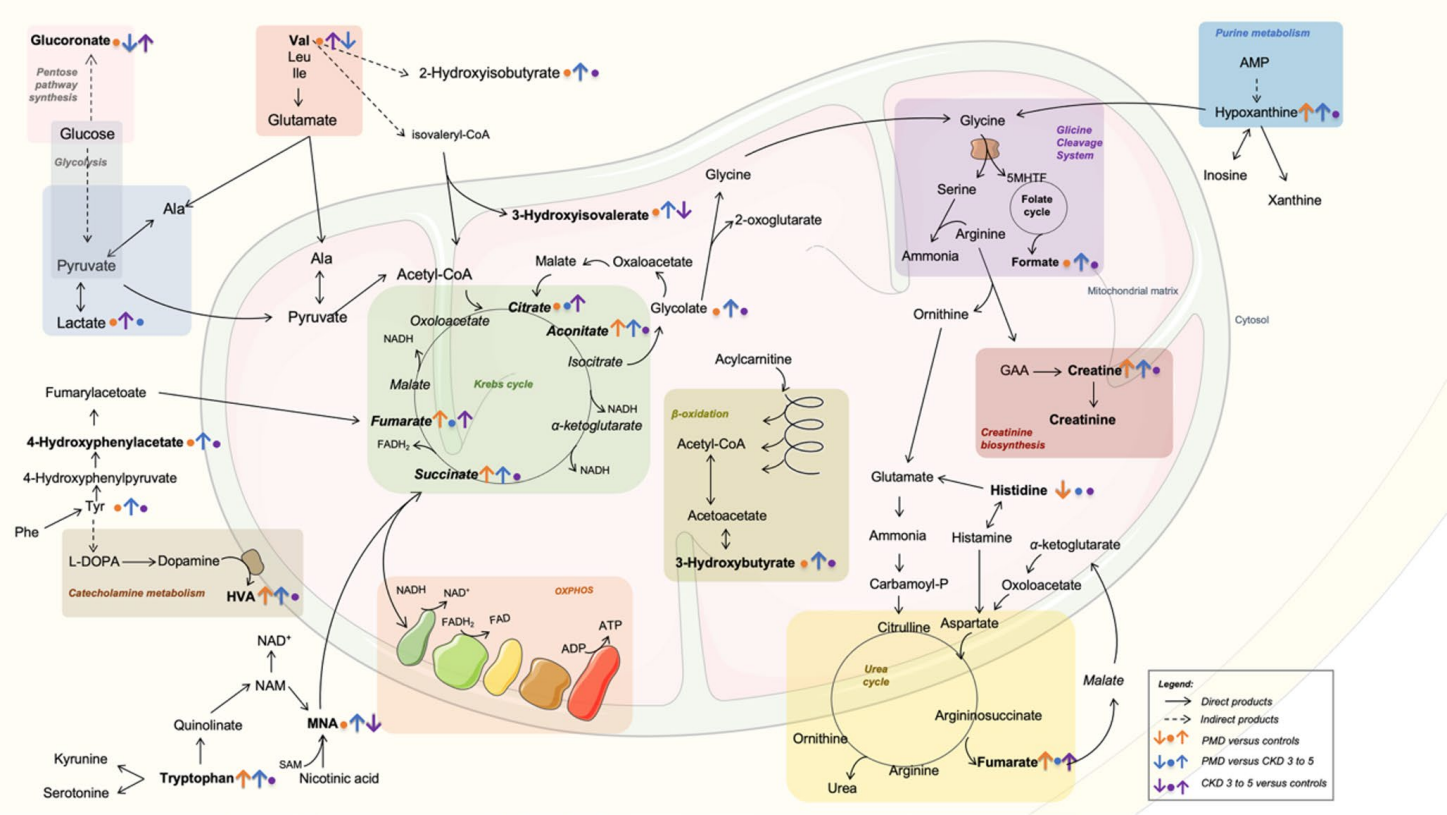
Overview of diverging metabolomic signatures between primary mitochondrial disease (PMD) and chronic kidney disease (CKD) and controls, based on significant urinary metabolites identified by ¹H NMR spectroscopy. Significance is based on univariate analysis. Arrows indicate the direction of change (↑ or ↓) or non-significant change (circle) in urinary levels in PMD versus controls (orange), PMD versus CKD stages 3–5 (blue), and CKD stages 3–5 versus controls (purple): This schematic representation of how pathways interact does not account for tissue variability (for example, urea cycle occurring predominantly in hepatocytes while creatine metabolism happens mainly in muscle and brain). Three-letter code used for amino acids. Adapted with partial use of graphical elements from Servier Medical Art (https://smart.servier.com), licensed under Creative Commons Attribution 3.0 *CKD* chronic kidney disease, *FAD* flavin adenine dinucleotide, *HVA* homovanillate, *L-DOPA* L-3,4-dihydroxyphenylalanine, *NAD⁺/NADH* nicotinamide adenine dinucleotide (oxidized/reduced), *OXPHOS* oxidative phosphorylation, *PMD* primary mitochondrial disease

Figure 5 illustrates the putative metabolic distinctions observed between PMD and CKD, highlighting key urinary metabolites altered in each condition and their integration within mitochondrial metabolic pathways.

These findings reinforce the pathobiological divergence between primary mitochondrial dysfunction and advanced CKD and reinforce the utility of urinary metabolomics in discriminating disease-specific biochemical signatures. Simultaneous, these results reinforce the value of disease-specific control groups in metabolomic studies as an opportunity for better understanding of disrupted metabolic pathways.

### Candidate biomarkers for CKD

Two potential biomarkers specific for CKD (but not PMD) were decreased MNA, and 3-hydroxyisovalerate, both of which effectively discriminated between CKD stage 3–5 and earlier stages and controls.

Previous studies that proposed MNA as a proxy for proximal tubular secretion (Maïza et al., 1992) and also demonstrated its reduction in tubulointerstitial disorders, such as Focal Segmental Glomerulosclerosis (Hao et al., 2013). Our findings with preserved MNA levels in the PMD group, align with the mechanistic model proposed by Takahashi et al. (2022) (Takahashi et al., 2022), in which reduced MNA levels reflect significant tubular damage and fibrosis, rather than early dysfunction.

Although 2-hydroxyisobutyrate pathways and renal metabolism are less well characterized, a previous metabolomic study associated its urinary excretion with early but not advanced diabetes-related CKD (Lucio-Gutiérrez et al., 2022). In our data, 2-hydroxyisobutyrate distinguished between early and advanced CKD, but not between CKD and control groups. Additionally, citrate excretion was significantly reduced only in advanced CKD (stages 3–5) as previously reported for diabetes-CKD(Kwan et al., 2020), supporting a progressive decline in urinary citrate with progressive renal impairment, possibly reflecting impaired distal nephron acid–base regulation in advanced CKD. MNA although not consistently altered in PMD versus controls, was strongly decreased in CKD3–5, contributing to discrimination between renal and mitochondrial signatures. These findings highlight the critical need to differentiate mitochondrial-specific metabolic derangements from renal-related metabolic changes when interpreting urinary biomarkers in clinical and research settings.

### Candidate biomarkers for PMD

Several metabolites emerged as potential urinary biomarkers for PMD, based on consistent alterations across multiple comparisons and their metabolic relevance. Increased tryptophan and decreased histidine emerged as the most specific discriminators of PMD versus both SMD) and controls. Succinate and fumarate, both Krebs cycle intermediates, were consistently elevated in PMD and SMD compared to controls, supporting their utility as sensitive—though not specific—markers of mitochondrial dysfunction. In contrast, cis-aconitate was significantly increased in PMD but reduced in advanced CKD, highlighting its potential to differentiate mitochondrial from renal dysfunction. Similarly, HVA was increased in both PMD and SMD and may reflect broader neuro-metabolic perturbations rather than PMD-specific alterations. Notably, the consistent decrease in urinary histidine in PMD, despite normal levels in SMD and early CKD, and its further reduction in advanced CKD, raises the hypothesis of subclinical renal involvement in PMD patients. This possibility warrants further investigation, as early mitochondrial-related renal dysfunction may contribute to altered amino acid excretion patterns.

HVA, the major dopamine catabolite, is generated via sequential metabolism by mitochondrial monoamine oxidase (MAO) and cytosolic catechol-O-methyltransferase (COMT), establishing a link between mitochondrial redox function and dopamine turnover (Naoi & Maruyama, 2010). HVA is excreted by glomerular filtration and tubular secretion, predominantly via OAT1 and OAT3 (Burckhardt et al., 2001; You et al., 2025) Urinary HVA has been validated as a useful surrogate of central dopaminergic metabolism in selected monogenic conditions, even when measured by non-targeted ¹H NMR approaches (Brennenstuhl et al., 2020). In our cohort, urinary HVA was elevated in PMD, suggesting alterations in mitochondrial-dependent monoamine metabolism or energy-coupled transport. Alterations in cerebrospinal HVA have been reported across several mitochondrial phenotypes and interpreted as secondary to impaired oxidative metabolism and MAO-dependent dopamine catabolism (Batllori et al., 2018; Papandreou et al., 2018). HVA was particularly elevated (> 2SD above control mean) in the patient with *TMEM70*-related disease, presenting with mild movement disorder sequelae following severe neonatal-onset. A 1.5-fold increase was also observed in patients with *m.8993T > G* (P11), *LYRM4* (P14), and *m.3271T > C* (P05) mutations. While the first has Leigh syndrome the others have no neurological or cognitive features. These findings raise the possibility of subclinical alterations in dopaminergic turnover or tissue-level oxidative metabolism, and underscore the potential of HVA as a sensitive, though nonspecific, marker of mitochondrial dysfunction even in the absence of a recognized neurological phenotype, as previously suggested in POLG-related disease and other mitochondrial disorders (Batllori et al., 2018; Papandreou et al., 2018).

Histidine was consistently decreased in PMD patients when compared to controls, suggesting a possible depletion or altered reabsorption in the proximal tubule. The reduction was more pronounced in PMD compared to CKD3-5, where a mild reduction was observed. Reduction of histidine in CKD rats was previously established (Zhang et al., 2015). As an essential amino acid, histidine participates in multiple mitochondrial-dependent processes, including oxidative stress defense, acid–base buffering, and the biosynthesis of carnosine and histamine (Brosnan & Brosnan, 2020; Li & Hoppe, 2023). Histidine catabolism occurs via mitochondrial pathways, involving its conversion to glutamate and subsequently to *α*-ketoglutarate. This conversion requires tetrahydrofolate (THF), directly linking histidine metabolism to one-carbon metabolism and mitochondrial energy production (Brosnan & Brosnan, 2020). Thus, the consistent reduction in urinary histidine in PMD may reflect increased mitochondrial utilization to replenish *α*-ketoglutarate pools or impaired energy-dependent renal reabsorption.

The consistent elevation of metabolites such as tryptophan and hypoxanthine in PMD patients, divergent from both controls and CKD, strongly suggests a distinct, mitochondria-driven metabolic phenotype rather than subclinical renal damage. Further studies are needed to determine whether these metabolomic signatures predict future renal involvement or represent stable, disease-specific traits associated with mitochondrial dysfunction.

Our findings underscore the potential of specific urinary metabolites, such as tryptophan and histidine, as novel candidate biomarkers for primary mitochondrial disease. Given the increasing number of clinical trials targeting mitochondrial dysfunction, the identification of robust and non-invasive biomarkers is essential to support patient stratification and treatment monitoring.

Building upon the consistent differences across pairwise comparisons, we identified a five-metabolite panel (*cis*-aconitate, fumarate, HVA, tryptophan and histidine) with high discriminatory power for PMD, which were subsequently validated trough multivariate modeling. These metabolites capture both PMD-specific alterations—notably increased tryptophan and reduced histidine—and broader mitochondrial dysfunction signatures, including elevated fumarate and cis-aconitate, which also contributed to separation from CKD patients. HVA further strengthened the panel’s ability to delineate PMD from overlapping phenotypes. Overall, this metabolic signature integrates specificity and sensitivity, supporting its potential use in clinical stratification and diagnostic workflows for suspected mitochondrial disease.

However, as previously highlighted, the development of such biomarkers remains a major challenge due to clinical and genetic heterogeneity, lack of standardization, and limited size of patient cohorts (Rahman & Rahman, 2018). Further validation in larger, longitudinal studies is needed to determine whether these metabolites can serve as biomarkers for supporting diagnosis or even surrogate endpoints in future therapeutic trials.

### Study limitations

Methodological limitations include a relatively small sample size (for instance adequate external validation of statistical models), absence of strict age and sex matching between groups, and significant genetic and clinical heterogeneity, particularly within the SMD and CKD cohorts. The cross-sectional design, based on single spot urine samples and non-standardized dietary conditions, may introduce biological and environmental variability. Although there were age differences between groups, previous studies suggest that age exerts limited impact on urinary metabolomic profiles in pediatric cohorts.

Similarly, while the absence of standardized fasting protocols introduces potential variability, it reflects real-world clinical practice. In this context, the metabolic signatures observed are likely to be persistent and disease-driven rather than transient nutritional artifacts. As previously recognized, fasting state represents a potential pre-analytical confounder in urinary metabolomic studies, however, while physiological variability was moderate among controls, greater dispersion was observed in PMD and CKD patients, likely reflecting disease-modified metabolic responses rather than nutritional variability.

Although ^1^H NMR is a robust and reproducible technique for untargeted analysis, it has lower sensitivity for detecting low abundance metabolites.

## Conclusions

This study demonstrates that untargeted urinary ^1^H NMR metabolomics is a feasible and informative approach for detecting metabolic alterations in pediatric primary mitochondrial disease We identified consistent disease-specific metabolic signatures, with significantly increased urinary levels of fumarate, *cis*-aconitate, HVA, hypoxanthine, tryptophan, and succinate, and decreased histidine in PMD compared to both controls and patients with CKD, even in the absence of renal impairment .

These findings support the translational potential of ^1^H NMR-based urinary metabolomics as a non-invasive platform for biomarker discovery for diagnostic and monitoring, patient stratification, and assessment of renal involvement in mitochondrial disease.

## Supplementary Information

Below is the link to the electronic supplementary material.

Supplementary Material 1

## Data Availability

The full processed dataset and raw 1 H NMR spectra have been deposited at the Metabolomics Workbench under Study ID ST003924 (Project DOI: http://dx.doi.org/10.21228/M81Z64).

## Abbreviations

¹H NMR: Proton nuclear magnetic resonance spectroscopy
ACR: Albumin-to-creatinine ratio
AKI: Acute kidney injury
AUC: Area under the curve
CAKUT: Congenital anomalies of the kidney and urinary tract
CKD: Chronic kidney disease
eGFR: Estimated glomerular filtration rate
FC: Fold change
GC-MS: Gas chromatography–mass spectrometry
HVA: Homovanillic acid (or homovanillate)
KEGG: Kyoto encyclopedia of genes and genomes
MA: Microalbuminuria
MNA: 1-methylnicotinamide
MVA: Multivariate statistical analysis
PCA: Principal component analysis
PLS-DA: Partial least squares–discriminant analysis
PMD: Primary mitochondrial disorder
SMD: Suspected mitochondrial disorder
SMPDB: Small molecule pathway database
UTI: Urinary tract infection
VIP: Variable importance in projection
VUR: Vesicoureteral reflux
