# Supplementary material for: NMR-based urinary biomarkers in pediatric primary mitochondrial disorders and chronic kidney disease: shared mitochondrial dysfunction, diverging biosignatures
Source: Metabolomics. 2026 Jan 19;22(1):17. doi: 10.1007/s11306-025-02363-8 (PMC12812765; doi:10.1007/s11306-025-02363-8)
Supplement: Supplementary file 1 — Supplementary Material 1 [file 11306_2025_2363_MOESM1_ESM.docx]

Supplementary Material

# NMR-based Urinary Biomarkers in Pediatric Primary Mitochondrial Disorders and Chronic Kidney Disease: Shared Mitochondrial Dysfunction, Diverging Biosignatures

Margarida Paiva Coelho^1,2,*^, João E. Rodrigues^3^, Teresa Costa^4^, Aureliano Dias^5^, Inês C.R. Graça^3^, Hugo Rocha^5,6^, Laura Vilarinho^5^, Esmeralda Martins^1,2^, Ana M. Gil^3*^

^1^Reference Center for Inherited Metabolic Disorders, Centro Hospitalar Universitário de Santo António, Unidade Local de Saúde de Santo António, 4099-001 Porto, Portugal

^2^Unit for Multidisciplinary Research in Biomedicine (UMIB)**,**School of Medicine and Biomedical Sciences (ICBAS), University of Porto, Rua Jorge Viterbo Ferreira 228, 4050-313 Porto, Portugal;

^3^Department of Chemistry and CICECO-Aveiro Institute of Materials, University of Aveiro, Campus Universitário de Santiago, 3810-193 Aveiro, Portugal

^4^Pediatric Nephrology Department, Centro Materno Infantil do Norte Albino Aroso, Centro Hospitalar Universitário de Santo António, Unidade Local de Saúde de Santo António, 4099-001 Porto, Portugal

^5^Newborn Screening, Metabolism and Genetics Unit, Human Genetics Department, National Institute of Health Doutor Ricardo Jorge, Portugal

^6^Department of Pathological, Cytological and Thanatological Anatomy, E2S, Polytechnic of Porto, Portugal

### *** Corresponding authors:**

Margarida Paiva Coelho, MD; [mmargaridacoelho.dca@chporto.min-saude.pt](mailto:mmargaridacoelho.dca@chporto.min-saude.pt)

Ana M. Gil: [agil@ua.pt](mailto:agil@ua.pt)

Figure S1. Recruitment flowchart.

Figure S2. Multivariate and univariate statistical analysis comparing primary mitochondrial disease (PMD) patients and controls.

Figure S3. Correlation analysis among urinary metabolites in PMD versus controls.

Figure S4. Metabolite correlation network based on Spearman’s rank among urinary metabolites in PMD versus controls

Figure S5. Quantitative enrichment pathway analysis of PMD versus controls.

Table S1. Detailed inclusion and exclusion criteria.

Table S2. Clinical and genetic characterization of groups.

Table S3. List of identified metabolites.

Table S4. Quality Parameters of PLS-DA models used to explore urinary metabolic profiles.

Table S5. List of significant metabolite variations for comparisons not included in Table 2.

Table S6. Spearman’s rank correlation coefficients for each quantified metabolite between Probabilistic Quotient Normalization (PQN) and creatine normalization.

### **Figure S1**

**Figure S1.  Recruitment Flowchart.** Flowchart of participant selection and exclusions during the recruitment process. Final groups used in the statistical analysis are shown in the bottom box, along with the corresponding number of patients and urine samples per group.

## **Figure S2**


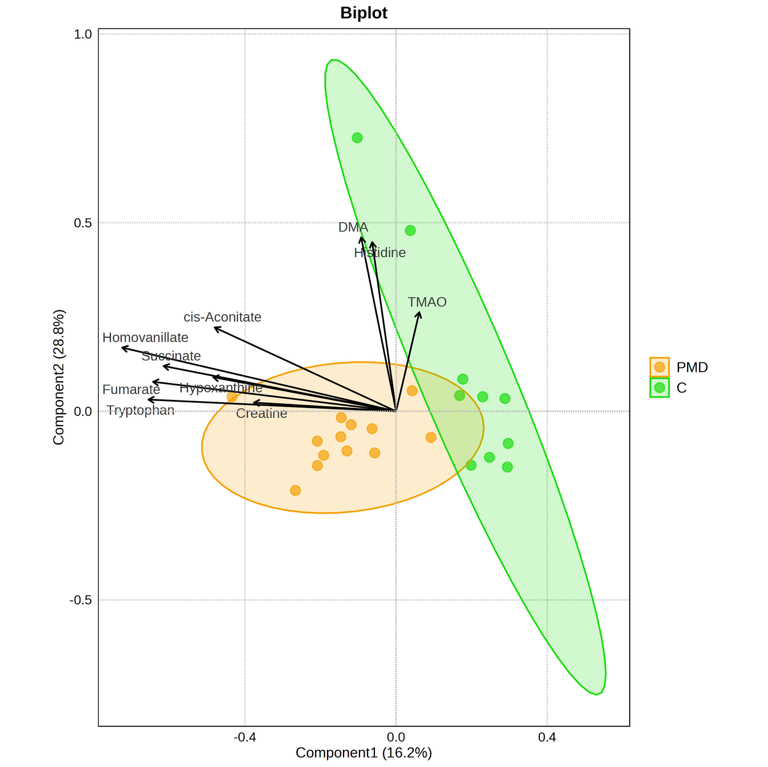


**A.**

**B.**

**C.**


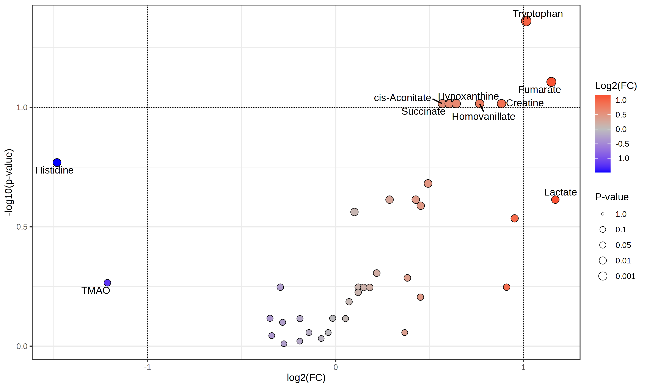

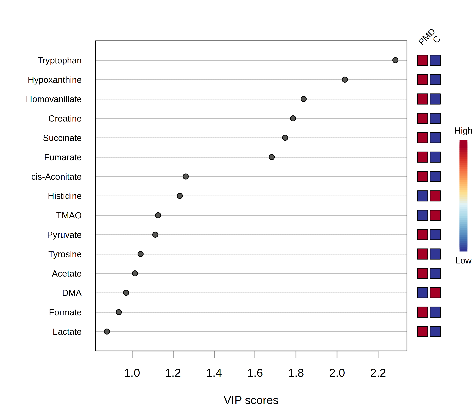


Figure S2. Multivariate and univariate statistical analysis comparing primary mitochondrial disease (PMD) patients and controls. A. PLS-DA biplot based on quantified metabolites (one peak per metabolite) illustrating the separation between PMD patients (orange) and controls (green). Arrows represent the direction and relative contribution of individual metabolites to group discrimination. B. Volcano plot showing the distribution of metabolites according to log₂ fold change (x-axis) and –log₁₀(p-value) (y-axis). Dotted lines indicate the thresholds for |log₂(FC)| > 1 and *p* < 0.05. C. Variable Importance in Projection (VIP) plot from the PLS-DA model, listing the top discriminating metabolites with corresponding heatmap profiles per group. All panels were generated using MetaboAnalyst 5.0 (https://www.metaboanalyst.ca).

**Figure S3**


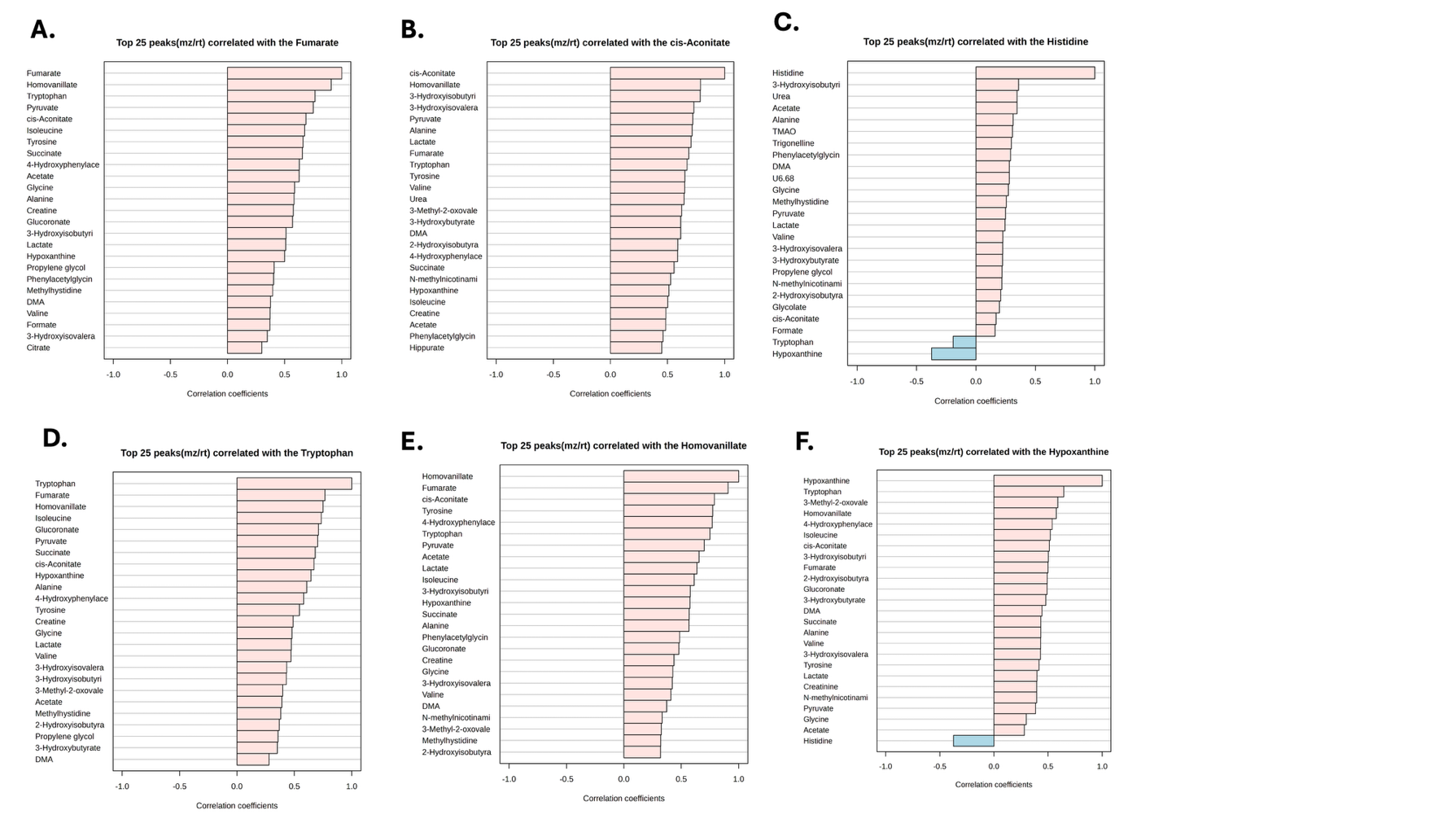


## **Figure S3. Metabolite correlation network based on Spearman’s rank among urinary metabolites in PMD versus controls, highlighting significant positive (red) and negative (blue) associations.** A–F. Top 25 metabolites most strongly correlated with fumarate (A), cis-aconitate (B), histidine (C), tryptophan (D), homovanillate (E), and hypoxanthine (F). Panels highlight consistent co-regulation among TCA cycle intermediates, amino acids, and redox-related metabolites. All analyses were performed using MetaboAnalyst 5.0 (https://www.metaboanalyst.ca) and R v4.4.0.

## **Figure S4**

**
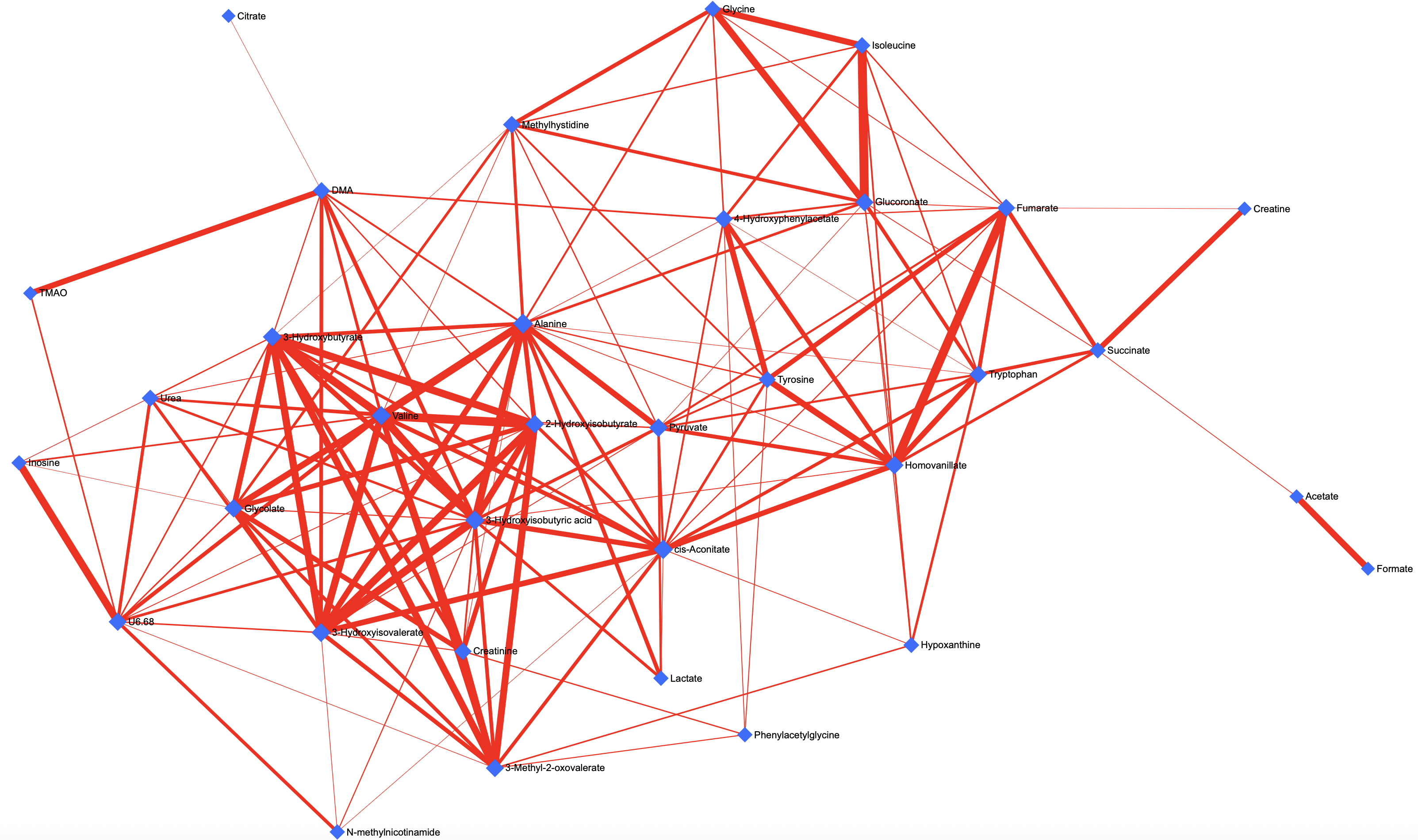
**

**Figure S4. Debiased Sparse Partial Correlation (DSPC) network of urinary metabolites in PMD.** This network illustrates partial correlations among urinary metabolites in patients with primary mitochondrial disease (PMD), computed using the DSPC algorithm implemented in MetaboAnalyst 5.0. Each node (blue diamond) represents an individual metabolite, and edges indicate statistically significant partial correlations after debiasing and shrinkage. Edge thickness is proportional to the strength of the association. Only the top-ranked 20% of edges (by p-value) are displayed for clarity. The input data were PQN-normalized and log-transformed prior to analysis. The network reveals clusters of tightly co-regulated metabolites, particularly within energy metabolism and branched-chain amino acid pathways. Topological hubs—such as valine, 3-hydroxyisobutyrate, alanine, DMA, glycine, and *cis*-aconitate—exhibited the highest degree of connectivity, suggesting their central role in the disrupted metabolic architecture observed in PMD.

## **Figure S5**

**
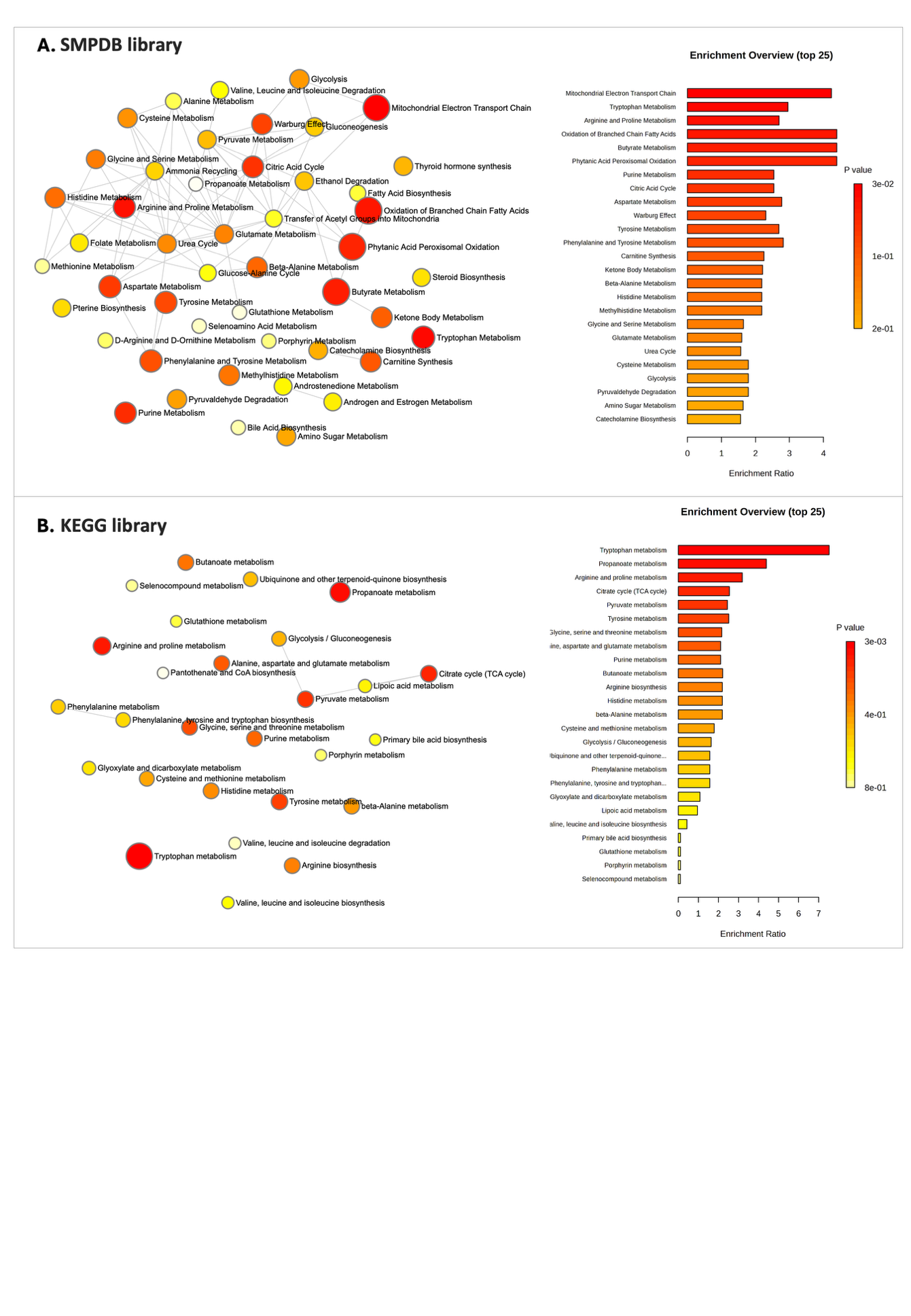
**

**Figure S5. Quantitative enrichment pathway analysis of PMD versus controls. A.** Enrichment results based on the Small Molecule Pathway Database (SMPDB) library; **B.** Enrichment results based on the Kyoto Encyclopedia of Genes and Genomes (KEGG) library. Analyses were performed using Quantitative Enrichment Analysis (QEA) in MetaboAnalyst 5.0. Pathways are ranked by adjusted p-value and enrichment ratio. Node color represents statistical significance (*p*-value), and node size represents pathway impact.

**Table S1.** **Detailed inclusion and exclusion criteria.** *Calculated by the creatinine-based Chronic Kidney Disease in Children (CKiD) U25 Equation except for patients younger than 12 months, where eGFR is based on the Schwartz formula, and for patients older than 18 years, where eGFR is based on CKD-EPI 2021 guidelines. Abbreviations: M: months; Y: years; CAKUT: Congenital Anomalies of the Kidney and Urinary Tract; eGFRcr: creatinine based estimated glomerular filtration rate; UTI: urinary tract infection.

| Group | Inclusion Criteria | Exclusion Criteria |
| --- | --- | --- |
| Applicable to all | Age >1M and <20 y | Active UTI |
| Primary mitochondrial disorders (PMD) | Genetically confirmed mitochondrial disease (nDNA or mtDNA) | Concomitant CKD  Acute intercurrent illness or metabolic decompensation at the time of sampling |
| Suspected mitochondrial disease (SMD) | Suspected PMD based on clinical/biochemical presentation without genetic confirmation | Other diagnoses confirmed by genetic studies |
| Chronic Kidney disease (CKD) | eGFRcr 90 mL/min/1.73m²* | Transplant recipient  CKD due to PMD |
| Control | Otherwise healthy patients with any mild condition (e.g. transitory creatinine elevation, CAKUT, or post-UTI follow-up) without evidence of CKD (eGFR; normal urinalysis, normal acid-base balance) | Other known organ/system involvement or development issues  Other suspected or confirmed (eg. genetically) conditions |
|  | | |

## **Table S2.** **Clinical and Genetic Characterization of Groups.** eGFR was calculated using age-appropriate creatinine-based equations: Schwartz formula (k = 0.45 if <1 year; k = 0.413 if 1–18 years) and CKD-EPI if >18 years. ***For autosomal diseases, the implicated gene is indicated, whereas for mtDNA, the associated variant is presented; ****Calculated by the creatinine-based Chronic Kidney Disease in Children (CKiD) U25 Equation except for patients younger than 12 months, where eGFR is based on the Schwartz formula, and for patients older than 18 years, where eGFR is based on CKD-EPI 2021 guidelines**; ***CKD stage based on eGFR and albumin-to-creatinine (ACR) ratio as defined by the KDIGO 2024 Guidelines. Abbreviations: 3-MGA-uria: 3-methylgluthaconic aciduria; ACR**: Albumin-to-creatinine ratio; **AD**: Autosomal Dominant; **AR**: Autosomal Recessive; **ASD**: Autism Spectrum Disorder; **CAKUT**: Congenital Anomalies of the Kidney and Urinary Tract; **DM**: Diabetes Mellitus; **FS**: Fanconi Syndrome; **FTT**: Failure to Thrive; **GRACILE**: Growth retardation, Renal (kidney) dysfunction, Aminoaciduria, Cholestasis, Iron overload, Lactate acidosis, Early death; **GDD**: Global Developmental Delay; in: intermittent; **LS**: Leigh Syndrome; MDC: mitochondrial disease criteria; **MMC**: Myelomeningocele; **M**: Male; **NS**: Nephrotic Syndrome; **NSHL**: Neurosensory Hearing Loss; OAs: organic acids chromatography; **PKD**: Polycystic Kidney Disease; Prot(u): proteinuria; SLSMD: single large-scale mtDNA deletions; **T1D**: Type 1 Diabetes; **UTI**: Urinary Tract Infection; **VUR**: Vesicoureteral Reflux;; **X-L**: X-linked; **y**: years; **F**: Female.

| Group | ID | Age (y) | Sex | Phenotype | Genetic basis* | Usual medication | eGFR ** | U25-Cr | U25-CisC | U25 Mean eGFR | ACR | CKD stage  ** | Prot (u) |
| --- | --- | --- | --- | --- | --- | --- | --- | --- | --- | --- | --- | --- | --- |
| PMD | P02 | 8.7 | F | Hypotonia; GDD; basal ganglia lesions | *EARS2* | Q10, thiamine, riboflavine | 194 | 165.1 | x | x | x | G1A1 | 0.187 |
|  | P03 | 18.8 | M | Primary Q10 deficiency | *COQ8A* | Clonazepam, ibedenone, Q10 | 105 | 82.42 | x | x | no | G1A1 | 0.036 |
|  | P04 | 14 | F | LS | m.10191T>C | Q10, thiamine, riboflavine | 124.8 | 116.6 | x | x | no |  | 0.09 |
|  | P05 | 10.5 | M | Cardiomiopathy, myopathy, NSHL, FFT | m.3271T>C | Q10, thiamine, riboflavine | 159.6 | 148.9 | x | x | no | G1A1 | 0.147 |
|  | P06 | 10.4 | M | LS | m.8993T>G | Q10, thiamine, riboflavine, arginine | 158.9 | 148.2 | x | x | 12.8 | G1A1 | 0.124 |
|  | P07 | 4.0 | F | FFT, Low stature, miopathy, muscle weakness, ptosis | SLSMD | Levotiroxine, Q10, thiamine, riboflavine, L-carnitine | 156.2 | 103.1 | x | x | x | G1A1 | x |
|  | P08 | 15.5 | F | LS | m.3946G>A | Q10, Thiamine, riboflavine | 91.36 | 86.47 | x | x | x | G1A1 | 0,072 |
|  | P09 | 4.8 | M | Cardiomiopathy intermittent 3-MGA | *TAZ* | Captopril, spironolactone, carvedilol | 129.1 | 115 | x | x | x | G1A1 | 0.172 |
|  | P10 | 1.0 | M | Neonatal severe metabolic acidosis, high Lactate, cardiomyopathy, 3-MGA-uria | *TMEM70* | Q10, thiamine, riboflavine, arginine, bicarbonate | 91.63 | 79.27 | x | x | 19.2 | G1A1 | 0.392 |
|  | P11 | 16.9 | F | LS | m.8993T>G | Q10, thiamine, riboflavine | 135.5 | 132.4 | x | x | x | G1A1 | in. |
|  | P12 | 1.1 | F | Alpers syndrome | *POLG* | Clonazepam, phenobarbital, lacosamide, Q10, thiamine, riboflavin | 334.5 | 268.1 | x | x | 26.1 | G1A1 | 0.522 |
|  | P13 | 8.8 | M | Epilepsy and ASD | m.8993T>C | Risperidone, atomexetine, valproate | 128.2 | 118 | x | x | x | G1A1 | 0.118 |
|  | P14 | 9.4 | F | Cardiorrespiratory arrests, 3-MGA-uria | *LYRM4* | None | 185.9 | 159.1 | x | x | 7.1 | G1A1 | x |
|  | P15 | 10.5 | F | GRACILE | *BCSL1* | Q10, thiamine, bicarbonate, phosphate , enalapril, esomeprazol, colecalciferol | 88.8 | 68.07 | 87.76 | 77.92 | 278.9 | G2A3 | x |
|  | P25 | 9.8 | M | NS, NSHL and myopathy | *RR2MB* | Q10, thiamine, steroids, micophenate mofetil, pravastatine | 83.25 | 77.37 | 41.7 | 59.53 | 9175 | G2A3 | 11,582 |
| SMD | P16 | 0.3 | M | ALTE, metabolic acidosis, ketosis, high alanine, low arginine, undiagnosed. MDC 5 | - | Arginine | 75.4 | x | x | x | x | G1A1 | x |
|  | P17 | 16.8 | F | Recurrent myositis, easy fatigability; CV 11%; MDC 2 | - | Q10 | 122 |  | x | x | x | G1A1 | x |
|  | P18 | 0.9 | F | Neonatal distress with hypoglycemia; early onset hypotonia; spasticity, assimetric ptosis, sensory disturbance; GDD; MDC 6 | - | None | 109 |  | x | x | x | G1A1 | x |
|  | P19 | 0.2 | F | Aregenerative anemia, neutropenia, ethylmalonic aciduria and 3-methylglutaconic aciduria (3MGA); neonatal distress without sentinel event; MDC 4 | - | Phenobarbital | 82.8 |  | x | x | x | G1A1 | x |
|  | P20 | 18.7 | F | FTT; LV hypertrophy cardiomiopathy without heart failure; muscle biopsy with multiple RC deficiencies; MDC =2 | - | Q10, thiamine, riboflavine | 136 | 118.5 | x | x | x | G1A1 | x |
|  | P21 | 3.7 | M | Progressive spasticity (++ lower limb), GDD; MDC=4 | - | Q10, thiamine, riboflavine | 125.3 |  | x | x | 13.8 | G1A1 | 0.13 |
|  | P22 | 4.5 | F | Episodic ataxia and sonnomlence; low stature; intermittent high Lactate and alanine; hyperintense cerebellar lesions. Krebs metabolites in OAs; MDC=5 | - | Q10, thiamine, riboflavine | 123.3 | 101.8 | x | x | 0 | G1A1 | 0 |
|  | P23 | 19.5 | F | Epileptic encephalopathy, absent language, GDD/ID; MDC=3 | - | Vigabatrine, zonisamide, lacosamide, ketogenic diet | 132 | 108.3 | x | x | 0 | G1A1 | 0 |
|  | P24 | 2.2 | F | FFT, low stature, muscle weakness, paradoxal ketosis, persistent high Lactate and alanine, distal tubular acidosis, Oas Krebs metabolites, MDC=7 | - | Q10, thiamine, riboflavine | 118.4 |  | x | x | <9 | G1A1 | 0.239 |
| C | P26 | 2.7 | M | CAKUTRenal duplication with bilateral pyelectasis | - | None | 122 | 108.1 | 96.6 | 102.4 | 8.3 | G1A1 | 0.194 |
|  | P28 | 3.1 | F | Previous UTI | - | None | 162.7 | 132.4 | 92.9 | 112.7 | 13.8 | G1A1 | 0.23 |
|  | P29 | 9.8 | M | Crossed renal ectopia | - | None | 129.1 | 111.9 | 106.1 | 109 | 9.9 | G1A1 | 0.1 |
|  | P30 | 13.3 | M | Ureterohydronephrosis | - | None | 102.9 | 106.1 | 91.8 | 98.95 | 6.3 | G1A1 | 0.114 |
|  | P31 | 12.4 | F | Renal duplication with bilateral pyelectasis | - | None | 109.7 | 100.4 |  |  | <3,4 | G1A1 | 0.063 |
|  | P32 | 8.1 | F | Vesicoureteral reflux | - | None | 122.8 | 104.8 | 80.6 | 92.7 | <3,4 | G1A1 | 0.125 |
|  | P33 | 13.5 | M | Vesicoureteral reflux with spontaneous regressions | - | None | 110.8 | 119.4 | 89.9 | 104.7 | 26.3 | G1A1 | 0.146 |
|  | P34 | 13.9 | M | Transient creatinine elevation | - | None | 100.1 | 107.9 | 90.8 | 99.35 | 7.4 | G1A1 | 0.06 |
|  | P35 | 18.2 | M | Posterior urethral valve (corrected) | - | None | 130 | 107.4 | 90.5 | 98.9 | 9.5 | G1A1 | 0.05 |
|  | P36 | 16.8 | M | Solitary kidney | - | None | 85.66 | 105.4 | 89.7 | 97.55 | 11.9 | G1A1 | 0.06 |
| CKD1-2 | P37 | 6.6 | M | Ataxia Telangiectasia | *ATM* | None | 112.5 | 102.9 |  |  | 16.9 | G1A1 | 0.15 |
|  | P38 | 15.8 | M | Polymalformative syndrome with CAKUT | - | None | 70.45 | 82.9 | 73.1 | 78 | 17.3 | G2A1 | 0.054 |
|  | P39 | 17.9 | F | CAKUT with multicystic kidneys | - | None | 113 | 83.3 | 84.1 | 83.7 | 3.1 | G2A1 | 0.053 |
|  | P40 | 14.3 | M | Renal Agenesia and ASD | - | None | 79.85 | 86 | 89.9 | 87.95 | 2.3 | G2A1 | 0.044 |
|  | P41 | 8.1 | F | PKD | - | None | 129.1 | 112.8 | 90.7 | 101.8 | 7.2 | G1A1 | 0.158 |
|  | P42 | 17.8 | M | Steroid-dependent NS | - | Steroids, Mycophenolate mofetil | 138 | 127 | 96.4 | 111.7 | 4.5 | G1A1 | 0.043 |
|  | P43 | 11.1 | M | X-L Hypophosphatemic Rickets (PHEX) | *PHEX* | Burosumab | 134 | 117.1 | 70.7 | 93.9 | 16.5 | G1A1 | 0.13 |
|  | P44 | 7.2 | M | X-L Hypophosphatemic Rickets | *PHEX* | Burosumab | 163 | 149.1 | 120.6 | 134.9 | 5.4 | G1A1 | 0.144 |
|  | P45 | 17.1 | M | Alport Syndrome | *COL4A3* | None | 88.87 | 113.2 | 87.6 | 100.4 | 14.4 | G1A1 | 0.064 |
|  | P46 | 4.8 | M | Steroid-sensitive NS; ASD | - | Steroids, Mycophenolate mofetil | 259.9 | 234 | X | X | 516 | G1A3 | 0.864 |
|  | P47 | 2.8 | F | VUR and UTIs ; T1D | - | Insuline | 207.4 | 101.3 | X | X | 2742 | G1A3 | 5.326 |
|  | P48 | 16.9 | M | Nutcracker Syndrome | - | 0 | 82.65 | 101.7 | x | X | 158.7 | G1A2 | 0.255 |
|  | P49 | 11.4 | M | Steroid-dependent NS | - | Steroids, cyclosporine, ramipril | 150.4 | 148.4 | X | X | 2020 | G1A3 | 2.419 |
|  | P50 | 14.8 | F | PKD | - | None | 105.2 | 100.8 | 73 | 86.9 | 79.3 | G1A2 | 0.161 |
|  | P51 | 16.2 | M | Probable IgA Nephropathy | - | Ramipril | 97.62 | 114.9 | 101.7 | 108.3 | 782.3 | G1A3 | 1.075 |
|  | P52 | 7.8 | M | Glomerulonephritis - IgA Nephropathy | - | Finerenone | 146.8 | 135.4 | X | x | 3174 | G1A3 | 4.11 |
|  | P53 | 17.3 | M | Urethral stricture | - | None | 87.58 | 107.7 | 87.6 | 97.65 | 148 | G1A2 | 0.386 |
| CKD3-5 | P54 | 12.9 | F | Cystinosis | *CTNS* | Cysteamine, phosphate, bicarbonate, sodium citrate, potassium citrate, potassium chloride, paricalcitol, calcium carbonate, colecalciferol, iron | 26.12 | 23.9 | 43.8 | 33.85 | 3983 | G4A3 | 6.242 |
|  | P55 | 4.7 | M | CAKUT with dysplastic and cystic kidneys | - | Bicarbonate, epoetin α, paricalcitol, colecalciferol, folic acid | 13.99 | 12.6 | 18.3 | 15.45 | 2650 | G5A3 | x |
|  | P56 | 15.7 | M | Ochoa syndrome with CAKUT | *HNF1B* | Calcium carbonate, amlodipine, colecalciferol, silodosin, resin, alfacalcidol, iron | 43.27 | 50.9 |  |  | 13.4 | G3aA1 | x |
|  | P57 | 9.5 | M | Joubert syndrome w/ nephronophthisis | *TCTN2* | Bicarbonate, iron, calcitriol, allopurinol, epoetin alfa, amlodipine, esomeprazole | 10.42 | 9.8 | 17.6 | 13.7 | 194.6 | G5A2 | x |
|  | P58 | 15.8 | M | CAKUT | - | Bicarbonate, calcitriol, enalapril, oxybutynin, tamsulosin, resin | 18.95 | 22.3 | 22.1 | 22.2 | 46.3 | G4A2 | x |
|  | P59 | 14.6 | M | AR-PKD and liver fibrosis | *PKHD1* | Bicarbonate, epoetin alfa, colecalciferol, paricalcitol, iron | 23.53 | 26.5 | 26.4 | 26.45 | 15.7 | G4A1 | x |
|  | P60 | 8.2 | M | Scarring nephropathy; VUR | - | Bicarbonate, epoetin alfa, colecalciferol, paricalcitol, enalapril, oxybutynin, somatotropin | 16.24 | 15.2 | 26.1 | 20.65 | 76.4 | G4A2 | x |
|  | P61 | 15.3 | F | MMC; VUR; neurogenic bladder | - | Bicarbonate, epoetin alfa, colecalciferol, paricalcitol, iron, calcium carbonate | 11.42 | 10.9 | 17.9 | 14.4 | 3063 | G5A3 | x |
|  | P62 | 10.8 | F | AR-PKD | *PKHD1* | Bicarbonate, iron, alfacalcidol, sevelamer, epoetin alfa, propranolol, calcium carbonate, colecalciferol | 14.74 | 12.8 | 19 | 15.9 | 75.9 | G5A1 | x |

**Table S3**. **List of identified metabolites**. All metabolites listed were assigned with a confidence level of Putative identified metabolite (MSI Level 2) according to Metabolomics Standards Initiative - MSI), except those marked with ^#^, tentative assignment. For each metabolite, the main metabolic pathway, HMDB and KEGG IDs, and spectral references are indicated. Peaks used for integration are shown in bold and underlined. Metabolites that were quantified are marked with a Y. Abbreviations: AA: amino acid; HMDB: Human Metabolome Database; KEGG: Kyoto Encyclopedia of Genes and Genomes. Multiplicity: s: singlet; d: doublet; dd: doublet of doublets; ddt: doublet of doublets of triplets; t: triplet; q: quartet; m: multiplet; br: broad signal.

| Compound Name | Main pathway | HMDB ID | KEGG ID | δ/ppm (multiplicity) | Quantified |
| --- | --- | --- | --- | --- | --- |
|  |  |  |  |  | (Y/N) |
| Phenylalanine | Aromatic AA metabolism | HMDB0000159 | C00079 | 3.19 (m); 4.00 (dd); 7.34 (m); 7.40 (m); 7.43 (m) | N |
| Alanine | Alanine metabolism | HMDB0000161 | C00041 | **1.48** (d); 3.78 (q) | Y |
| Dimethylamine (DMA) | Amine metabolism | HMDB0000087 | C00543 | **2.71** (s) | Y |
| Acetate | Beta-oxidation/ketone metabolism | HMDB0000042 | C00033 | **1.92** (s) | Y |
| Acetoacetate | Beta-oxidation/ketone metabolism | HMDB0000060 | C00164 | 2.27 (s); 3.42 (s) | N |
| Acetone | Beta-oxidation/ketone metabolism | HMDB0001659 | C00207 | 2.23 (s) | N |
| 3-Methyl-2-oxovalerate (3M2OV) | Branched-chain AA metabolism | HMDB0000491 | C00671 | **0.89** (d); 1.10 (d); 2.20 (m); 2.55 (m) | Y |
| Isoleucine | Branched-chain AA metabolism | HMDB0000172 | C00407 | 0.94 (t); **1.02** (d); 1.28 (m); 1.46 (m); 1.99 (m); 3.67 (d) | Y |
| Leucine | Branched-chain AA metabolism | HMDB0000687 | C00123 | **0.97** (t); 1.71 (m); 1.74 (m); 3.74 (t) | Y |
| Valine | Branched-chain AA metabolism | HMDB0000883 | C00183 | 0.99 (d); **1.04 (**d); 2.27 (m); 3.62 (d) | Y |
| α-Glucose | Carbohydrate metabolism | HMDB0003345 | C00031 | 3.41 (m); 3.54 (dd); 3.72 (t); 3.77 (t); 3.83 (m); 5.23 (d) | N |
| β-Glucose | Carbohydrate metabolism | HMDB0000122 | C00221 | 3.24 (dd); 3.41 (m); 3.47 (m); 3.50 (t); 3.73 (dd); 4.65 (d) | N |
| Glucose-1-phosphate ^#^ | Carbohydrate metabolism | HMDB0001586 | C00103 | 3.37 (t); 3.46 (m); 3.48 (m); 3.70 (d); 3.89 (m); 5.51(dd) | N |
| *myo*-inositol | Carbohydrate metabolism | HMDB0000211 | C00137 | 3.26 (m); 3.52 (m); 3.61 (m); 3.64 (m) | N |
| Creatine | Creatine metabolism | HMDB0000064 | C00300 | 3.04 (s); **3.93** (s) | Y |
| Creatinine | Creatine metabolism | HMDB0000562 | C00791 | 3.05 (s); **4.06** (s) | Y |
| Homovanillate (HVA) | Dopamine metabolism | HMDB0000118 | C05582 | 3.43 (s); 6.76 (d); **6.94** (dd); 7.10 (d) | Y |
| Ethanol | Ethanol metabolism | HMDB0000108 | C00469 | 1.17 (t); 3;65 (q) | N |
| Glucuronate | Glucoronate pathway | HMDB0000127 | C00191 | 3.30 (dd); 3.50 (m); 3.57 (dd); 4.09 (d); 4.65 (d); **5.27** (d) | Y |
| Glycine | Glycine and serine metabolism | HMDB0000123 | C00037 | **3.57** (s) | Y |
| *cis*-Aconitate | Glycolysis/Krebs cycle | HMDB0000072 | C00417 | **3.11** (d); 5.70 (s) | Y |
| Citrate | Glycolysis/Krebs cycle | HMDB0000094 | C00158 | 2.52 (dd); **2.67** (dd) | Y |
| Fumarate | Glycolysis/Krebs cycle | HMDB0000134 | C00122 | **6.52** (s) | Y |
| Malonate | Glycolysis/Krebs cycle | HMDB0000691 | C00383 | 3.11 (s) | N |
| Oxoglutarate | Glycolysis/Krebs cycle | HMDB0000208 | C00026 | 2.45 (t); 3.00 (s) | N |
| Pyruvate | Glycolysis/Krebs cycle | HMDB0000243 | C00022 | **2.38** (s) | Y |
| Succinate | Glycolysis/Krebs cycle | HMDB0000254 | C00042 | **2.41** (s) | Y |
| Glycolate | Glyoxylate and dicarboxylate metabolism | HMDB0000115 | C03547 | **3.96** (s) | Y |
| 5-Aminolevulinate (5-ALA) | Heme biosynthesis | HMDB0001149 | C00430 | 2.42 (dt); 2.51 dt); 2.74 (t); 3.81 (s) | N |
| 1-Methylhistidine | Histidine metabolism | HMDB0000001 | C01152 | 3.34 (s); **7.03** (s); 8.15 (s) | Y |
| Histidine | Histidine metabolism | HMDB0000177 | C00135 | 3.15 (dd); 3.24 (dd); 3.98 (t); **7.10** (s); 8.08 (s) | Y |
| 3-Hydroxybutyrate (3-HBA) | Ketone metabolism | HMDB0000011 | C01089 | **1.23** (d); 2.24 (dd); 2.39 (dd); 4.15 (m) | Y |
| 3-Hydroxyisovalerate (3-HIVA) | Leucine metabolism | HMDB0000754 | C20827 | **1.27** (s); 2.35 (s) | Y |
| 2-Aminoadipate | Lysine degradation | HMDB0302754 | C00956 | 1.63 (m); 1.81 (m); 2.07 (m); 2.23 (t); 2.33 (m); 3.86 (m) | N |
| Lysine | Lysine degradation | HMDB0000182 | C00047 | 1.47 (m); 1.75 (m); 3.03 (m) | N |
| Trimethylamine *N*-oxide (TMAO) | Methylamine metabolism | HMDB0000925 | C01104 | **3.27** (s) | Y |
| Betaine | Methylation metabolism | HMDB0000043 | C00719 | 3.26 (s); 3.90 (s) | N |
| Trigonelline | Nicotinate metabolism | HMDB0000875 | C01004 | 4.43 (s); 7.11 (d); 8.07 (d); 8.85 (s); **9.13** (s) | Y |
| Formate | One carbon pool by folate | HMDB0000142 | C00058 | **8.46** (s) | Y |
| *N*-Phenylacetylglycine (*N*- PhAcGly) | Phenylalanine metabolism | HMDB0000821 | C05598 | 3.64 (s); 3.82 (s); **7.36** (m); 7.43 (m) | Y |
| Hypoxanthine | Purine metabolism | HMDB0000157 | C00262 | **8.19** (s); 8.22 (s) | Y |
| Inosine ^#^ | Purine metabolism | HMDB0000195 | C00294 | 3.86 (dd); 3.93 (dd); 4.34 dd);4.38 (td); 4.78 (dd); 6.09 (d); 8.23 (s); **8.34** (s) | Y |
| Lactate | Pyruvate metabolism | HMDB0000190 | C00186 | **1.34** (d); 4.11 (q) | Y |
| *N*-Methylnicotinamide (MNA) | Nicotinate and nicotinamide metabolism pathway | HMDB0003152 | NA | 4.48 (s); 8.18 (m); 8.90 (d); 8.97 (d); **9.29** (s) | Y |
| 2-Hydroxyisobutyrate (2-HIBA) | Short-chain fatty acid metabolism | HMDB0242161 | NA | 1.26 (s); **1.36** (s) | Y |
| Methionine | Sulfur-containing AA metabolism | HMDB0000696 | C00073 | 2.10 (s); 2.65 (t); 3.88 (t) | N |
| Taurine | Sulfur-containing AA metabolism | HMDB0000251 | C00245 | 3.27 (t); 3.43 (t) | N |
| Threonine | Threonine metabolism | HMDB0000167 | C00188 | 1.32 (d); 4.22 (m) | N |
| 4-Hydroxyphenylacetate (4-HPA) | Tyrosine / Phenylalanine metabolism | HMDB0060390 | C13636 | 3.54 (s), 6.87 (d), **7.17** (d) | Y |
| Tyrosine | Tyrosine metabolism | HMDB0000158 | C00082 | 3.06 (dd); 3.19 (dd); 3.93 (dd); **6.90** (d); 7.20 (d) | Y |
| Tryptophan | Tryptophan metabolism | HMDB0000929 | C00078 | 7.29 (t); 7.55 (d); **7.75** (d) | Y |
| 3-Indoxylsulfate ^#^ | Tryptophan metabolism | HMDB0000682 | NA | 7.21 (dd); 7.28 (dd); 7.36 (s); 7.51 (d); 7.70 (d) | N |
| Urea | Urea cycle | HMDB0000294 | C00086 | **5.83** (br, s) | Y |
| 3-Hydroxyisobutyrate (3-HIBA) | Valine metabolism | HMDB0000023 | C06001 | **1.11** (d); 2.65 (m); 3.52 (m); 3.68 (m) | Y |
| 3-Aminoisobutyrate (BAIBA) | Valine metabolism | HMDB0003911 | C05145 | **1.20** (d); 3.55 (m); 3.01 (dd); 3.10 (dd) | Y |
| Hippurate | Xenobiotic metabolism | HMDB0000714 | C01586 | 3.96 (d); 7.54 (m); 7;63 (tt) **7.83** (dd); 8.52 (br) | Y |
| Propylene glycol ^#^ | Xenobiotic metabolism/Possible contaminant | HMDB0001881 | C00583 | **1.14** (d); 3.43 (dd); 3.53 (dd); 3.86 m | Y |
| Tartrate ^#^ |  | HMDB0000956 | C00898 | 4.35 (s) | N |

**Table S4.** **Quality Parameters of PLS-DA models used to explore urinary metabolic profiles.** Each row corresponds to a PLS-DA model comparing specific diagnostic or phenotypic subgroups based on UV-scaled urinary ^1^H NMR spectra. R²X and R²Y represent the fraction of variance explained by the model in the predictors (X) and response (Y) matrices, respectively. Q² corresponds to the predictive ability of each model as estimated by 7-fold cross-validation. Models with Q² values above 0.5 (shown in bold) were considered to have moderate to good predictive performance. Subgroup validation was performed for the presence of albumin-to-creatinine ratio (ACR >30mg/mg creatinine) in the CKD1-2 group, without discrimination between both subsets. Notably, comparisons between PMD and CKD subgroups Abbreviations: PMD: Primary Mitochondrial Disease; SMD: Secondary Mitochondrial Dysfunction; CKD: Chronic Kidney Disease; MA Microalbuminuria (defined by ACR >30mg/mg creatinine).

| PLS-DA models | R^2^X | R^2^Y | Q^2^ |
| --- | --- | --- | --- |
| PMD x C | 0.18 | 0.96 | **0.53** |
| PMD x CKD1-2 | 0.15 | 0.96 | **0.62** |
| PMD x CKD3-5 | 0.27 | 0.97 | **0.78** |
| PMD x SMD | 0.17 | 0.93 | 0.25 |
| SMD x CKD1-2 | 0.17 | 0.94 | 0.45 |
| SMD x CKD3-5 | 0.31 | 0.97 | **0.85** |
| SMD x C | 0.18 | 0.97 | 0.45 |
| CKD1-2-MA x CKD1-2+MA | 0.20 | 0.95 | 0.29 |
| CKD1-2 x C | 0.16 | 0.88 | < 0 |
| CKD3-5 x C | 0.31 | 0.98 | **0.83** |
| CKD3-5 x CKD1-2 | 0.27 | 0.95 | **0.75** |

**Table S5.** **List of significant metabolite variations for comparisons not included in Table 2.** ^1^ Statistical significance (Sign.): **p*<0,05; ** *p*<0,01; *** *p*<0,001. Abbreviations: CKD: chronic kidney disease; FC: fold change; PMD: primary mitochondrial disease; SMD: suspected mitochondrial dysfunction; CKD: chronic kidney disease.

| Compound name | ppm | FC | Hedge’s (g) | *p*-value | Sign ^1^ |
| --- | --- | --- | --- | --- | --- |
| CKD1-2 vs Control |  |  |  |  |  |
| 4-Hydroxyphenylacetate | 6.89 | 1.48 | 0.42 | 0.033 | * |
| *cis*-Aconitate | 3.11 | 1.78 | 0.66 | 0.017 | * |
| 1-Methylhystidine | 7.03 | 2.94 | 0.86 | 0.015 | * |
| Tryptophan | 7.75 | 1.96 | 0.84 | 0.033 | * |
| CKD3-5 vs CKD1-2 |  |  |  |  |  |
| 2-Hydroxyisobutyrate | 1.36 | 0.99 | 0.01 | 0.005 | ** |
| 3-Hydroxybutyrate | 1.23 | 0.86 | 0.13 | 0.008 | ** |
| 3-Hydroxyisovalerate | 1.27 | 0.55 | 0.48 | 0.001 | *** |
| 3-Methyl-2-oxovalerate | 1.10 | 1.33 | -0.28 | 0.007 | ** |
| *cis*-Aconitate | 3.11 | 0.78 | 0.10 | 0.018 | * |
| Citrate | 2.67 | 0.92 | 0.10 | 0.000 | *** |
| Formate | 8.46 | 1.21 | -0.18 | 0.024 | * |
| Glucuronate | 5.27 | 6.69 | -5.61 | 0.004 | ** |
| Glycolate | 3.96 | 1.15 | -0.13 | 0.011 | * |
| Histidine | 7.10 | 0.80 | 0.22 | 0.010 | ** |
| Homovanillate | 6.94 | 0.89 | 0.10 | 0.003 | ** |
| Hypoxanthine | 8.19 | 0.50 | 0.56 | 0.015 | * |
| Lactate | 1.34 | 4.79 | -2.53 | 0.013 | * |
| 1-Methylhystidine | 7.03 | 0.84 | 0.13 | 0.041 | * |
| *N*-Methylnicotinamide | 9.29 | 0.62 | 0.39 | 0.001 | *** |
| *N*-Phenylacetylglycine | 7.36 | 0.71 | 0.32 | 0.007 | ** |
| Propylene glycol | 1.14 | 0.58 | 0.37 | 0.020 | * |
| Tyrosine | 6.90 | 0.96 | 0.04 | 0.001 | ** |
| Propylene | 1.14 | 0.58 | 0.37 | 0.020 | * |
| PMD vs CKD1-2 |  |  |  |  |  |
| 2-Hydroxyisobutyrate | 1.36 | 0.73 | 0.63 | 0.045 | * |
| Fumarate | 6.53 | 1.83 | -0.82 | 0.019 | * |
| Histidine | 7.10 | 0.40 | 1.20 | 0.001 | *** |
| *N-*Phenylacetylglycine | 7.36 | 0.60 | 0.62 | 0.045 | * |
| Valine | 1.05 | 0.69 | 0.85 | 0.027 | * |
| PMD vs SMD |  |  |  |  |  |
| 3-Hydroxybutyrate | 1.23 | 1.23 | 0.30 | 0.045 | * |
| Histidine | 7.10 | 0.50 | -1.49 | 0.005 | ** |
| Propylene glycol | 1.14 | 1.69 | 0.70 | 0.028 | * |
| SMD vs Control |  |  |  |  |  |
| Acetate | 1.93 | 1.13 | 0.12 | 0.030 | * |
| *cis*-Aconitate | 3.11 | 1.77 | 0.84 | 0.016 | * |
| Fumarate | 6.53 | 1.99 | 0.76 | 0.025 | * |
| Homovanillate | 6.94 | 1.46 | 0.18 | 0.006 | ** |
| Hypoxanthine | 8.19 | 1.44 | 0.25 | 0.002 | ** |
| Pyruvate | 2.38 | 1.60 | 0.66 | 0.037 | * |
| Succinate | 2.41 | 1.12 | 0.11 | 0.008 | ** |

**Table S6.** **Spearman’s rank correlation coefficients for each quantified metabolite between Probabilistic Quotient Normalization (PQN) and creatine normalization.**

| Metabolite | Spearman | p-value |
| --- | --- | --- |
| Trigonelline | 0.750 | <0.001 |
| Creatine | 0.715 | <0.001 |
| Citrate | 0.712 | <0.001 |
| Formate | 0.702 | <0.001 |
| Fumarate | 0.670 | <0.001 |
| TMAO | 0.643 | <0.001 |
| Propylene glycol | 0.643 | <0.001 |
| Hippurate | 0.606 | <0.001 |
| Phenylacetylglycine | 0.587 | <0.001 |
| Acetate | 0.576 | <0.001 |
| Glucoronate | 0.569 | <0.001 |
| Succinate | 0.569 | <0.001 |
| Homovanillate | 0.558 | <0.001 |
| Hypoxanthine | 0.555 | <0.001 |
| 4-Hydroxyphenylacetate | 0.526 | <0.001 |
| N-methylnicotinamide | 0.512 | <0.001 |
| Tryptophan | 0.487 | <0.001 |
| Histidine | 0.479 | <0.001 |
| Lactate | 0.476 | <0.001 |
| Pyruvate | 0.467 | <0.001 |
| Tyrosine | 0.440 | 0.001 |
| Isoleucine | 0.427 | 0.001 |
| *cis*-Aconitate | 0.419 | 0.001 |
| Methylhystidine | 0.407 | 0.002 |
| Glycine | 0.388 | 0.003 |
| Alanine | 0.315 | 0.016 |
| 3-Hydroxyisovalerate | 0.309 | 0.018 |
| Inosine | 0.250 | 0.059 |
| 2-Hydroxyisobutyrate | -0.188 | 0.157 |
| U6.68 | 0.184 | 0.167 |
| Valine | 0.167 | 0.211 |
| Urea | 0.164 | 0.219 |
| 3-Hydroxyisobutyric acid | 0.089 | 0.507 |
| DMA | 0.056 | 0.678 |
| Glycolate | -0.038 | 0.780 |
| 3-Hydroxybutyrate | 0.022 | 0.870 |
| 3-Methyl-2-oxovalerate | -0.018 | 0.891 |
